# Cox process representation and inference for stochastic reaction–diffusion processes

**DOI:** 10.1038/ncomms11729

**Published:** 2016-05-25

**Authors:** David Schnoerr, Ramon Grima, Guido Sanguinetti

**Affiliations:** 1 School of Biological Sciences, University of Edinburgh, Edinburgh EH9 3JH, UK; 2 School of Informatics, University of Edinburgh, Edinburgh EH8 9AB, UK; 3 SynthSys, University of Edinburgh, Edinburgh EH9 3JD, UK

## Abstract

Complex behaviour in many systems arises from the stochastic interactions of spatially distributed particles or agents. Stochastic reaction–diffusion processes are widely used to model such behaviour in disciplines ranging from biology to the social sciences, yet they are notoriously difficult to simulate and calibrate to observational data. Here we use ideas from statistical physics and machine learning to provide a solution to the inverse problem of learning a stochastic reaction–diffusion process from data. Our solution relies on a non-trivial connection between stochastic reaction–diffusion processes and spatio-temporal Cox processes, a well-studied class of models from computational statistics. This connection leads to an efficient and flexible algorithm for parameter inference and model selection. Our approach shows excellent accuracy on numeric and real data examples from systems biology and epidemiology. Our work provides both insights into spatio-temporal stochastic systems, and a practical solution to a long-standing problem in computational modelling.

## Abstract (web summary)

Stochastic reaction-diffusion systems are used for modelling spatial dynamics in many disciplines, but parameter inference and model selection remain challenging. Here the authors offer a solution enabled by a connection between reaction-diffusion and the well-studied spatio-temporal Cox processes.

## Graphical abstract

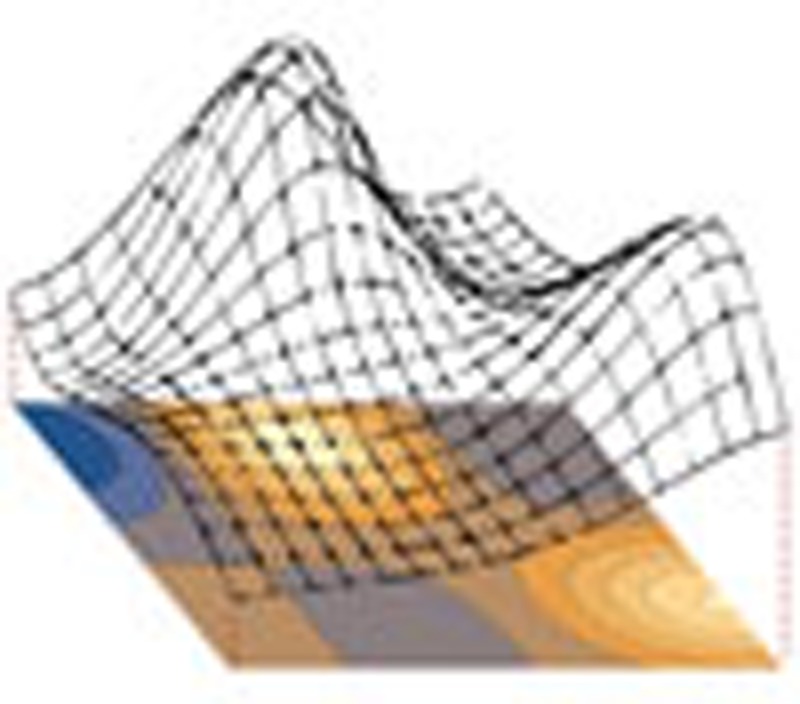

Many complex behaviours in several disciplines originate from a common mechanism: the dynamics of locally interacting, spatially distributed agents. Examples arise at all spatial scales and in a wide range of scientific fields, from the microscopic interactions of low-abundance molecules within cells, to ecological and epidemic phenomena at the continental scale. Frequently, stochasticity and spatial heterogeneity play a crucial role in determining the process dynamics and the emergence of collective behaviour^1^,^2^,^3^,^4^,^5^,^6^,^7^,^8^.

Stochastic reaction–diffusion processes (SRDPs) constitute a convenient mathematical framework to model such systems. SRDPs were originally introduced in statistical physics^9^,^10^ to describe the collective behaviour of populations of point-wise agents performing Brownian diffusion in space and stochastically interacting with other, nearby agents according to predefined rules. The flexibility afforded by the local interaction rules has led to a wide application of SRDPs in many different scientific disciplines where complex spatio-temporal behaviours arise, from molecular biology^4^,^11^,^12^, to ecology^13^, and to the social sciences^14^.

Despite their popularity, SRDPs pose considerable challenges, as analytical computations are only possible for a handful of systems^8^. Thus, many analytical techniques that are widely used for non-spatial stochastic systems cannot be used for SRDPs. From the practical point of view, perhaps the single most important outstanding problem is inference in SRDP models: given observations of the system, can we reconstruct the interaction rules/local dynamic parameters? Solving this inverse problem would be important, as it would allow to quantitatively assess model fit to data and to compare different models/hypotheses in the light of observations.

SRDPs are generally analysed by either Brownian dynamics simulations of individual particles or by resorting to spatial discretization, leading to the so-called ‘reaction–diffusion master equation' (RDME)^15^,^16^. The computational complexity of the RDME obviously increases as the spatial discretization becomes finer, and in many cases the limiting process does not lead to the original SRDP^17^. Significant effort has been spent to improve the performance of the two types of simulations^18^,^19^,^20^,^21^,^22^,^23^,^24^; however, the computational costs are still high and quickly become prohibitive for larger systems. More importantly, the lack of an analytical alternative to simulations means that evaluating the fit of a model to observations (the likelihood function) is computationally extremely expensive, effectively ruling out statistical analyses. As far as we are aware, the few attempts at statistical inference for SRDPs either used simulation-based likelihood free methods^13^, inheriting the intrinsic computational difficulties discussed above, or abandoned the SRDP framework by adopting a coarse space discretization^25^ or neglecting the individual nature of agents using a linear-noise approximation^26^.

In this paper, we propose an approximate solution to the problem of computing the likelihood function in SRDPs, thus providing a principled solution to the inverse problem of model calibration. Using the classical theory of the Poisson representation (PR) for stochastic reaction processes^27^, we show that marginal probability distributions of SRDPs can be approximated in a mean-field sense by spatio-temporal Cox point processes, a class of models widely used in spatio-temporal statistics^28^. Cox processes model the statistics of point patterns via an unobserved intensity field, a random function that captures the local mean of the observed point process. This relationship between SRDPs and Cox processes is surprising, as SRDPs are mechanistic, microscopic descriptions of spatio-temporal systems, while Cox processes are employed phenomenologically to explain regularities in point patterns. This novel link between these two classes of models enables us to formally associate an SRDP with a continuous evolution equation on the local statistics of the process in terms of (stochastic) partial differential equations (SPDEs). Crucially, this novel representation of SRDPs allows us to efficiently approximate multiple-time marginals and thus data likelihoods associated with observed point patterns, enabling us to leverage the rich statistical literature on spatio-temporal point processes for parameter estimation and/or Bayesian inference^28^,^29^.

We demonstrate the efficiency and accuracy of our approach for the problem of parameter inference and model selection by means of a number of numerical and real data examples, using non-trivial models from systems biology and epidemiology. Our results provide both a valuable resource for performing statistical inference and assessing model fit in this important class of models, and a novel conceptual perspective on spatio-temporal stochastic systems.

## Results

### SRDPs and the PR

In the classical Doi interpretation^9^,^10^, which we adopt here, SRDPs describe the evolution of systems of point particles performing Brownian diffusion in a spatial domain 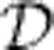. While SRDPs are used in a variety of disciplines, we will use the language of chemical reactions to describe them in the following. We assume the existence of *N* different types of particles *X*_*i*_, or species, which can interact through a set of predefined rules or reactions. We assume the structure of the model, that is, which reactions are possible, to be known exactly; later, we will relax this assumption to allow for the existence of a (finite) number of possible alternative mechanisms. Each particle of a particular species *X*_*i*_ performs Brownian diffusion with a species-specific microscopic diffusion constant *D*_*i*_. Bimolecular reactions between particles occur with a certain rate whenever two particles are closer than some specified reaction range. In principle, both reaction and microscopic diffusion rate constants may be space dependent, for example, to account for geometric constraints; for simplicity, we will only describe the homogeneous case here.

SRDPs are frequently analysed via coarse graining by discretizing space and assuming dilute and well-mixed conditions in each compartment; in this case the dynamics in each compartment is described by the chemical master equation^30^. Modelling diffusion of particles between neighbouring compartments as unimolecular reactions leads to the RDME^15^, which describes the dynamics of a continuous-time Markov jump process. For systems with only zero- or first-order reactions, the RDME converges to Brownian dynamics in the continuum limit. For non-linear systems, however, this is not the case in two or more dimensions because the rate of bimolecular reactions converges to zero, independently of the scaling of the corresponding rate constant^17^.

Consider now a set of chemical species *X*_*i*_ in a finite volume divided into *L* cubic compartments of edge length *h*, interacting via the following *R* reactions:


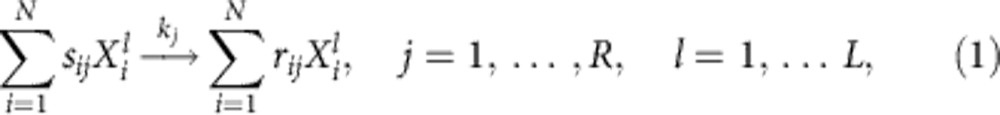



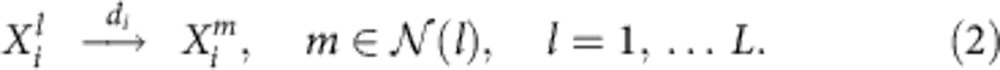


Here, *s*_*ij*_ and *r*_*ij*_ are the number of reactants and product particles of species *X*_*i*_ in the *j*th reaction, respectively, *k*_*j*_ is the rate constant of the *j*th reaction, 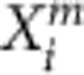 denotes species *X*_*i*_ in the *m*th compartment and *d*_*i*_ is the diffusion rate constant of species *X*_*i*_. The latter is related to the microscopic diffusion constant *D*_*i*_ via *d*_*i*_=*D*_*i*_/*h*^2^. We assume homogenous diffusion here, that is, *d*_*i*_ is independent of the compartment position, but it would be straightforward to extend the following analysis to space-dependent diffusion. 
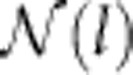
 denotes all the adjacent compartments of the *l*th compartment. Equation (1) describes chemical reactions happening in single compartments, whereas equation (2) describes diffusion between adjacent compartments. We confine our analysis to reactions with at most two reactant and at most two product particles, that is, 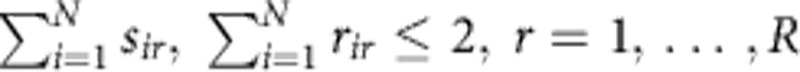, since higher-order reactions rarely occur in nature. We call a reaction linear if 
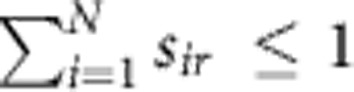
 and bimolecular if 
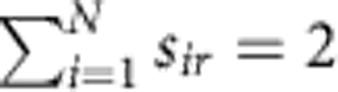
. Under well-mixed and dilute conditions in each compartment, the evolution of marginal probabilities of this system is given by the RDME that is given in general form in the Methods section.

In the case of only a single compartment, that is, *L*=1, the state of the system is given by **n**=(*n*_1_,…,*n*_*N*_), where *n*_*i*_ is the number of *X*_*i*_ particles in the system, and the time evolution of the single-time probability distribution *P*(**n**,*t*) is determined by the chemical master equation (see the Methods section). Gardiner derived an alternative description of the dynamics of such a system by making the ansatz of writing *P*(**n**,*t*) as a Poisson mixture^27^,^31^:


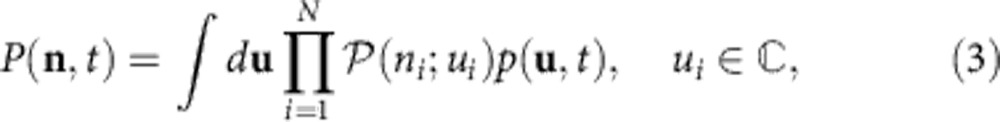


where **u**=(*u*_1_,…,*u*_*N*_) and 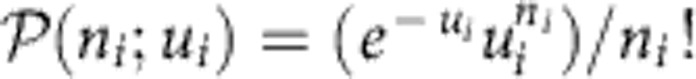 is a Poisson distribution in *n*_*i*_ with mean *u*_*i*_, and the *u*_*i*_ are complex-valued in general. Using this ansatz in the chemical master equation one can derive an exact Fokker–Planck equation for *p*(**u**,*t*) or equivalently a Langevin equation for **u**(*t*)^27^ (see Methods section for more details). Gardiner derived this result for the non-spatial chemical master equation and applied it to the RDME to study the corresponding continuum limit. While the PR provides an elegant analytical tool to study reaction systems, its applicability is severely hindered by the fact that the Poisson variables *u* are in general complex valued and hence lack a clear physical interpretation; in particular, all bimolecular reactions and all linear reactions with two non-identical product particles give rise to a complex-valued PR (for a taxonomy of which reaction systems become complex valued, see Supplementary Methods).

### Cox process representation

While in the classical view of the PR, the auxiliary variables *u*_*i*_ are simply introduced as a mathematical device, we can make some progress by considering a joint process over the *u*_*i*_ and *n*_*i*_ variables. Formally, this is equivalent to what in statistics is called demarginalization: a complex process is replaced by a (simpler) process in an augmented state space, such that the marginals of the augmented process return exactly the initial process. To formalize this idea, we first introduce some concepts from spatial statistics.

**Figure 1:**
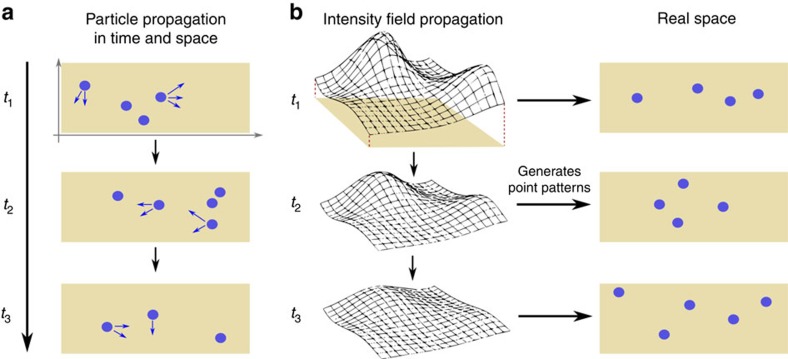
Visualization of Cox process approximation of SRDPs for multi-time points. (**a**) Time evolution of the true SRDP in space. Particles diffuse in space, may decay or are created and react with each other. (**b**) Time evolution of a Cox process. Here, the intensity field evolves in time, rather than the points in real space. The latter are merely noisy realizations of the intensity field. In particular, the point patterns at two different time points are independent of each other conditioned on the intensity field.

A (spatial) Poisson process^32^ is a measure on the space of zero-dimensional subsets of a domain 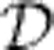; in this work, we consider Poisson processes that admit an intensity function *u*(*x*), which gives the rate of finding a point in an infinitesimally small spatial region. The number of points in a finite spatial region is then a Poisson random variable, with mean given by the integral of the intensity function over that region. A Cox process (also called ‘doubly stochastic Poisson process') is a generalization of a Poisson process, where the intensity field is itself a random process. Conditioned on a realization of the intensity field, the Cox process reduces to a Poisson process (see the Methods section for a more detailed definition of Poisson and Cox processes). We will consider families of spatial Poisson (Cox) process indexed by a time variable; importantly, in this case the intensity field can be thought of as the state variable of the system, with the actual spatial points being noisy realizations of this state (see Fig. 1 for a graphical explanation). Our first observation follows directly from Gardiner's analysis of the continuum limit of the RDME (see Supplementary Methods for a proof).

### Remark 1

Consider an SRDP on a spatial domain 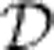 and temporal domain 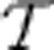, and let all reactions involve production or decay of at most one particle. Then, for appropriate initial conditions, 
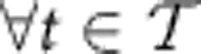
 the single-time-point spatial probability distribution of the SRDP is exactly the same as of a spatial Poisson process.

### General SRDPs

We can build on this point process representation to develop novel, mathematically consistent approximation schemes for SRDPs in general. Consider, for example, a bimolecular reaction of the type 
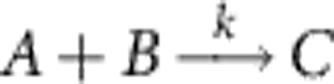
 with propensity function *f*(*n*_*A*_,*n*_*B*_)=*kn*_*A*_*n*_*B*_/Ω, where *n*_*A*_ and *n*_*B*_ are the number of *A* and *B* particles in the system, respectively, and Ω is the system volume. While the PR for such systems is complex valued, we can formally obtain a real system by applying a mean-field approximation that replaces the bimolecular reaction 
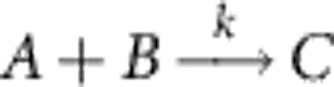
 by the two reactions 
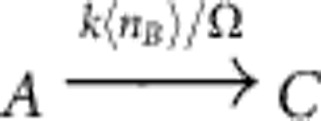
 and 
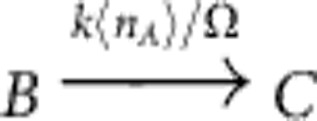
 with propensity functions *f*(*n*_*A*_,*n*_*B*_)=*kn*_*A*_〈*n*_*B*_〉/Ω and *f*(*n*_*A*_,*n*_*B*_)=*kn*_*B*_〈*n*_*A*_〉/Ω, respectively. Here, 〈·〉 denotes the expectation of a random variable with respect to its marginal distribution. The proposed approximation hence replaces the direct interaction of the particles by an effective interaction of *A* with the mean field of *B* and vice versa. Other bimolecular reactions and linear reactions with two non-identical product particles can be approximated in a similar manner. This leads to a real-valued evolution equation for the *u*_*i*_ variables, see Methods and Supplementary Methods for details and examples.

Applying this approximation to a general RDME, and subsequently the PR and taking the continuum limit gives the following set of *N* coupled SPDEs (see Methods section for a derivation)


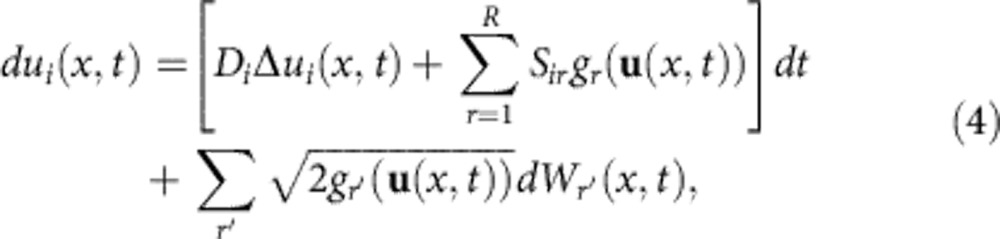


where the sum over *r*′ runs over all reactions with two product particles of species *X*_*i*_. In particular, this means that in the absence of reactions with two identical product molecules the diffusion term in equation (4) vanishes and equation (4) reduces to a partial differential equation (PDE), that is, the *u*_*i*_(*x*,*t*) are deterministic. *x* in equation (4) is a spatial location, *D*_*i*_=*h*^2^*d*_*i*_ is the microscopic diffusion constant of species *X*_*i*_, Δ is the Laplace operator, **u**(*x*,*t*)=(*u*_1_(*x*,*t*),…,*u*_*N*_(*x*,*t*)), where *u*_*i*_(*x*,*t*) is the intensity field of species *X*_*i*_, *dW*_*r*′_(*x*,*t*) is spatio-temporal Gaussian white noise and we have defined the propensity functions *g*_*r*_(**u**(*x*,*t*)) in PR space. The latter are obtained by applying the mean-field approximation to the propensity functions *f*_*r*_(**n**) and subsequently replacing *n*_*i*_→*u*_*i*_(*x*,*t*) and 〈*n*_*i*_〉→〈*u*_*i*_(*x*,*t*)〉. Note that the latter denotes a local expectation of the stochastic random field *u*_*i*_(*x*,*t*), rather than a spatial averaging. See Methods and Supplementary Methods for more details and examples.

To obtain equation (4), we approximated bimolecular reactions by linear reactions. Note, however, that the propensity functions of the latter reactions depend on the mean fields of certain species. This means that the resulting SPDEs in equation (4) are generally non-linear and hence in principle are able to capture non-linear dynamical behaviours.

Equation (4) looks similar to the spatial chemical Langevin equation^33^, but has a different interpretation here, since it describes the intensity in PR space. In particular, just as any other PDE or SPDE description in real space, the spatial chemical Langevin equation does not provide a generative model for the actual location of the events, and thus would not allow us to directly model statistically particle locations. Notice that, as a consequence of the mean-field approximation, the mean values of the *u*_*i*_ fields are the same as in a deterministic rate equation description; however, the dynamics of the observed variable, that is, the points in space, remain stochastic even when the intensity field evolves deterministically. We can therefore extend Remark 1 to obtain the following result (see Supplementary Methods for a proof).

### Result 1

Consider the same setting as in Remark 1. Under appropriate initial conditions, if there is at least one linear reaction with two product particles of the same species, the system's single-time-point distribution is exactly the same as of a Cox process, whose intensity fulfils the SPDE given in equation (4). If the system involves other types of reactions, including bimolecular reactions, the single-time probability distribution of the SRDP is approximated in a mean-field sense by that of a Poisson (Cox) process, whose intensity fulfils equation (4).

### The likelihood function

Result 1 provides an efficient means to calculate statistics such as expected number of agents within a certain volume, without the need to perform extensive Monte Carlo simulations, since it only requires to solve a (S)PDE for which a rich literature of numerical methods exists^28^,^29^. The numerical methods used in this paper are presented in Supplementary Methods. Importantly, we can use Result 1 to approximate the likelihood function of a configuration of points arising from an SRDP, by using the well-known Cox process likelihood: if *u*(*x*,*t*) is the intensity of a spatio-temporal Cox process with distribution *p*(*u*(*x*,*t*)) and **y** a given measurement at time *t*_0_, the corresponding likelihood is given by^28^


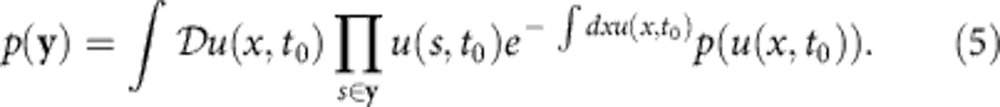


This function can be easily optimized to yield statistical estimates of kinetic parameters from single-time observations.

### Remark 2

We would like to emphasize that in the case of a Cox process, that is, a stochastic intensity field, the number of particles in two non-overlapping spatial regions are correlated random variables in general. The reason is that the PR ansatz in equation (3) is not merely a product of Poisson distributions, but rather an integral over such a product weighted by a corresponding mixing distribution. In the case of a Poisson process, that is, a deterministic intensity field, in contrast, the numbers of particles in two non-overlapping spatial regions are always uncorrelated.

### Time-series observations

We next consider the problem of approximating the joint distribution of point patterns arising from an SRDP at different time points. This is important when we have time-series observations, that is, spatial measurements 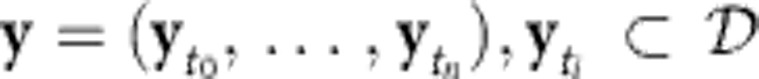 at discrete time points *t*_0_,…,*t*_*n*_, and we want to compute the likelihood *p*(**y**|Θ) of the data given a model Θ. Since the system is Markovian the likelihood factorizes as


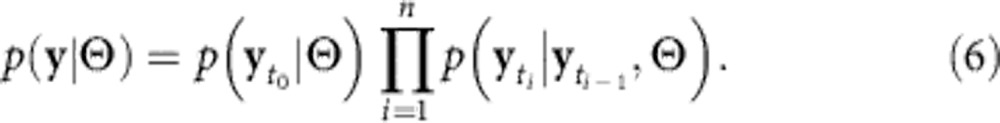


We would like to approximate this likelihood, using the relation to Cox processes established in Result 1. While this is in principle straightforward, computing the terms 
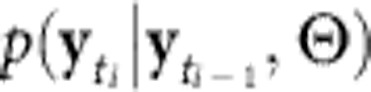
 involves determining the distribution over the associated *u*_*i*_(*x*,*t*) fields in PR space. This would involve inverting the PR transformation in equation (3), which is computationally inconvenient. Instead, we opt for an approximation strategy: assume that we have determined the PR distribution 
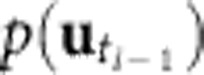
 at time *t*_*i*−1_, where we introduced the shorthand 
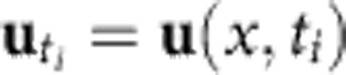
. By definition of the intensity of a Poisson process, 
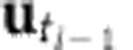
 represents the expectation of the random configuration of points 
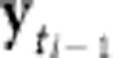
 at time *t*_*i*−1_. We then approximate 
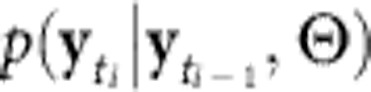
 in a mean-field way by replacing the explicit dependence on 
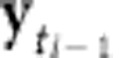
 with its expectation 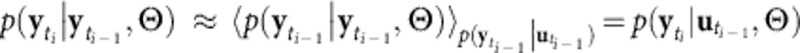. Figure 1 visualizes this approximation. Figure 1a shows the time evolution in an SRDP, while Fig. 1b shows the time evolution of a corresponding approximating Cox process. This leads to a new interpretation of the measured points 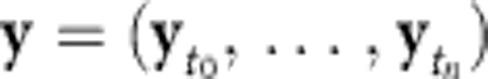: while they are snapshots of the actual state in the true system, they correspond to the noisy realizations of the state **u**(*x*,*t*) in the Cox process picture. We thus have the following result.

### Result 2

The joint *n*-time-point marginal distribution of an SRDP can be approximated in a mean-field sense by the joint probability of a Poisson (Cox) process, with intensity governed by the (S)PDE in equation (4).

### Relation to Gardiner's work

As mentioned before, Gardiner had already derived similar equations as equation (4) for single-time marginals of SRDPs^27^. Crucially, however, an approximation scheme for multiple-time joint marginals was, to our knowledge, never proposed; multi-time joint marginals are necessary for inference from time-series observations, hence the importance of Result 2. Furthermore, Gardiner's approach generally leads to a complex-valued PR; this motivates the novel approximation schemes of certain reactions that we introduced here. It is this novel real-valued PR, together with the interpretation of the PR variables as state variables, which allows us to derive the novel relation between SRDPs and spatio-temporal Poisson (Cox) processes.

### Inference

Result 2 is particularly powerful statistically, because it enables us to analytically approximate the exact (intractable) likelihood *p*(**y**|Θ) in equation (6), by the likelihood of a spatio-temporal Cox process with intensity *u*(*x*,*t*). The intensity itself follows the dynamics imposed by the PR in equation (4); importantly, the PR explicitly links the dynamic parameters governing the evolution of the intensity function to the microscopic diffusion and reaction rate constants of the SRDP.

Parameter estimation can therefore be performed efficiently by maximizing the Cox process likelihood. In the simpler case where the intensity function evolves deterministically, the likelihood can be evaluated numerically via the solution of a system of PDEs, and the dynamic parameters can be numerically recovered using standard optimization algorithms. In the case where the intensity function evolves stochastically, we evaluate the likelihood by an approximate filtering approach, as commonly used in many statistical and engineering applications (see Supplementary Methods for algorithmic details).

The availability of a likelihood function enables us to provide a statistically meaningful, data-driven assessment of how well a model describes the data. This is particularly important when there is uncertainty as to the precise mechanism underlying the data, for example, the exact reactions or species involved. Likelihood estimates, appropriately penalized to account for model complexity, can then be used to select models according to their support from the data.

It is important to notice that our approach directly optimizes the kinetic parameters of the model, rather than fitting an intensity function to the observed points and then fitting the dynamics. Since kinetic parameters are usually much fewer than the number of observations available, the risk of over-fitting is generally low in our approach.

Next, we apply Result 1 and Result 2 to several examples, and perform parameter inference by maximizing the data likelihood. We solve the corresponding (S)PDEs numerically by projecting them onto a finite set of spatial basis functions, see Supplementary Methods for details. The software used is available as Supplementary Data 1–4.

### Parameter estimation for a gene expression system

**Figure 2:**
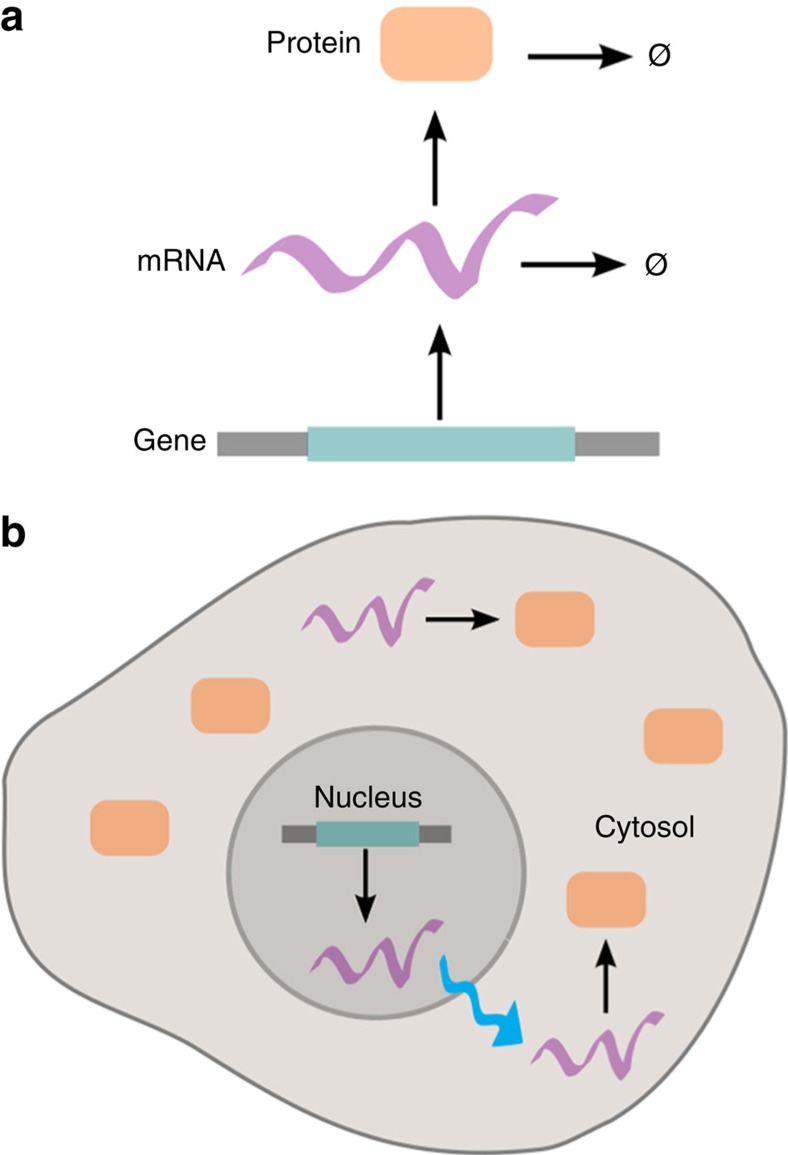
Gene expression system. (**a**) Chemical reactions taking place. (**b**) Illustration of system. The mRNA becomes transcribed in the nucleus, and becomes translated to proteins in the cytosol. mRNA and protein molecules decay stochastically and undergo Brownian diffusion across the whole cell.

To demonstrate the accuracy of our method, we first consider simulated time-series data in this section. Consider a gene expression system as illustrated in Fig. 2. For simplicitly, we consider a one-dimensional version of this system here, with the nucleus located at one side of the cell. A gene located in the nucleus is transcribed into messenger RNA (mRNA) molecules. The latter decay and diffuse across the whole cell and are translated into proteins in the cytosol. The protein molecules also diffuse across the whole cell and decay. For simplicity, we do not model the gene explicitly, but assume that mRNA becomes transcribed with a certain fixed rate *m*_1_ homogeneously in the nucleus. The corresponding reactions are


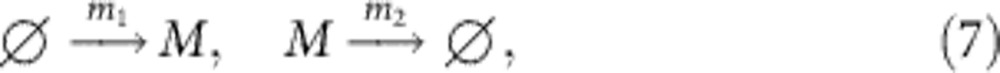



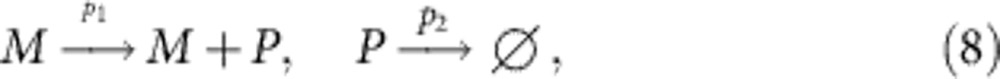


where *M* and *P* denote the mRNA and protein, respectively. For this system, the SPDE of our method in equation (4) becomes deterministic and thus corresponds to a Poisson process.

In addition to the reaction parameters *m*_1_, *m*_2_, *p*_1_ and *p*_2_, we have to infer the nucleus size *r*, as well as the diffusion rates *d*_m_ and *d*_p_ of the mRNA and protein, respectively, summing up to a total number of seven parameters. We assume that the positions of the protein molecules are observed at 30 time points, while the mRNA is unobserved. The results for one parameter set are shown in Table 1. Considering that we observe the protein at only 30 time points with unobserved mRNA and that we have seven unknown parameters, the inferred average values are remarkably close to the exact values. Moreover, the s.d.'s of the inferred results for single data sets are small, demonstrating the accuracy and precision of our method.

Next, we extend the system in Fig. 2 by adding an autocatalytic reaction for the protein,


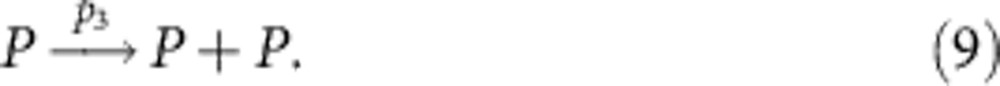


Including this reaction leads to a non-vanishing noise term in equation (4) and the system corresponds to a Cox process. We note that the system has a steady state only if *p*_3_<*p*_2_, with an otherwise exponentially growing mean protein number. On the mean level only the difference *p*_2_−*p*_3_ is identifiable, and we fix *p*_3_=0.01. We thus infer the same parameters as in the previous case, but this time modelled as a Cox process. Table 2 shows the results, indicating the accuracy of our method. See Supplementary Methods for more information on the used equations and algorithmic details.

### Parameter estimation for an SIRS model

We next consider the SIRS system, a popular model for describing the dynamics of an infection spreading through a population. Such systems are frequently modelled as SRDPs^34^ or discretized versions thereof^35^. We consider a system in the two-dimensional square [0,1] × [0,1]. The system comprises a susceptible (*S*), an infected (*I*) and a recovered species (*R*), which perform Brownian diffusion and interact via the reactions


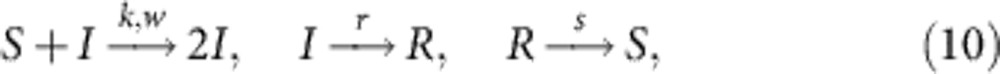


where the bimolecular infection is characterized by the microscopic reaction rate *k* and the reaction range *w*. We assume that all three species diffuse with the same diffusion rate *d*. We assume further that initially there are no recovered (*R*) particles, *S*_ini_ susceptible (*S*) particles placed uniformly over the whole area and one infected (*I*) particle located at [0.05, 0.05]. We consider the case that all three species are observed and perform inference for 40 simulated data points using Result 2, thereby replacing *k* and *w* by an effective bimolecular reaction parameter *k*^PR^. The model thus has four parameters that need to be inferred: the diffusion rate *d*, the recovery rate *r*, the susceptible rate *s* and the bimolecular infection rate *k*^PR^. Table 3 shows the corresponding results, demonstrating the accuracy and precision of our method. The computational efficiency of our method in comparison with stochastic simulations is particularly pronounced here. For the first parameter set in Table 3, for example, the Brownian dynamics simulation of a single realization of the system takes ∼250 s, while the whole inference procedure for the four parameters using our method takes only ∼10 s for one simulated data set on a 3.1 GHz core. See Supplementary Methods for more details.

**Figure 3:**
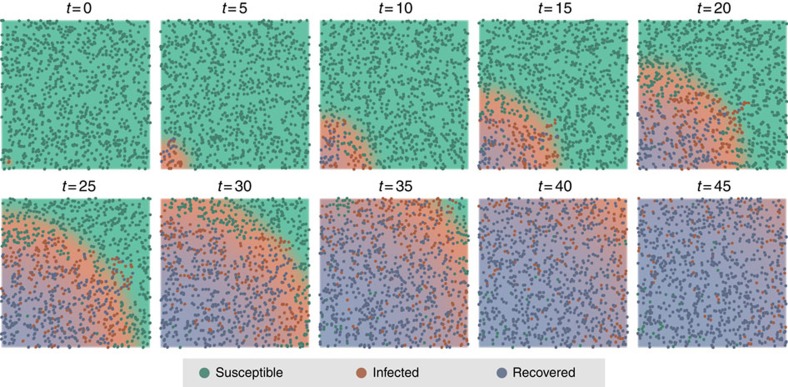
Dynamics of SIRS system. The figure shows the time evolution of a single simulated realization (points) and of the prediction of our method (background colours) for the SIRS system with reactions in equation (10) for time *t*=0–45, with steps of Δ*t*=5. For the simulation, we use the parameters (*S*_ini,_*k*, *w*, *d*, *r*, *s*)=(10^3^, 10^4^, 0.02, 0.0002, 0.3, 0.01) and for the point process prediction the corresponding inferred parameters. The background is an RGB image with the three colour components being proportional to the intensity fields of the three species *S* (turquoise), *I* (bronze) and *R* (blue). Notice how the mean-field approximation captures the complex behaviour of a wave of infection, spreading through the domain from the bottom left corner.

Figure 3 visualizes the dynamics of the SIRS system for one parameter set. Individual points from a simulation are shown in different colours (turquoise for S, bronze for I and blue for R), while the background colours represent a superposition of the respective intensity fields with optimized parameters. Notice how the PR approximation is able to capture the emerging behaviour of a wave of infection sweeping through the domain from bottom left to top right, before the establishment of a dynamic equilibrium between the three populations. Such a phenomenon is clearly due to the spatial aspect of the system, and could not have been recovered using an inference method that does not incorporate spatial information. Indeed, the overall number of infected individuals rises rapidly and remains essentially constant between time 20 and 35 before dropping to steady state, a behaviour that is simply not possible in a non-spatial SIRS model.

### Parameter estimation for *Drosophila* embryo data

**Figure 4:**
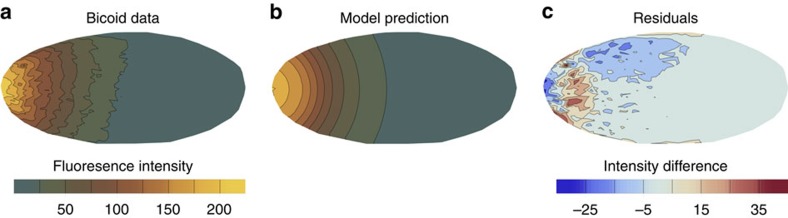
Results for the *Drosophila* embryo bicoid data. (**a**) Measurement data of bicoid fluorescence intensity across a single embryo. (**b**) Corresponding prediction of our point process model. (**c**) Difference of the experimental data and point process prediction. We observe the point process prediction agrees well with the experimental data. The point process prediction is obtained by solving equation (4) numerically for the inferred parameter values maximizing the data likelihood.

Finally, we apply our method to the real gene expression data for the bicoid protein at cleavage stage 13 in the *Drosophila* embryo. The data for 17 embryos can be obtained from the FlyEx database^36^. The data consists of fluorescence intensity measurements on a spatial grid and is shown for one embryo in Fig. 4a. The protein becomes expressed in some region at the left end of the embryo, and then diffuses across the embryo and decays. The system is typically modelled by a linear birth–death process^25^,^26^, and we assume the protein to be expressed within a certain distance *r* from the left end of the embryo. At cleavage stage 13, the system is supposed to be in steady state and we can perform inference using Result 1 and equation (4). For simplicity, we project the data to one dimension (see Supplementary Methods for more details).

The system has four parameters: the creation range *r*, the diffusion rate *d*, the production rate *c*_1_ and decay rate *c*_2_ of the bicoid protein. For steady-state data not all parameters, but only certain ratios are identifiable. We thus infer the creation range *r*, the diffusion rate *d* and the ratio *c*=*c*_1_/*c*_2_. For the average of the inferred parameters and their s.d.'s (shown here in parentheses) across the ensemble of embryos we obtain


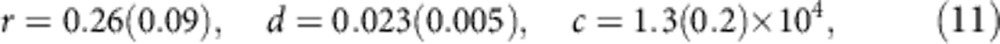


with s.d.'s of ∼20–30%. We find that these results do not change significantly under variations of the initial parameter values used in the likelihood optimiser.

Figure 4 illustrates the inference result for one embryo. Figure 4a,b shows the bicoid density across the whole embryo for experimental data and the PR prediction, respectively. We observe good agreement between the measurement data and the point process approximation. Figure 4c shows a plot of the model residuals (difference between model predictions and real data); as can be seen, these are generally comparatively small. As could be expected, the larger errors are concentrated around the steeply changing gradient region between the anterior segments and the main body of the embryo.

### Model selection for an SIRS model

Next, we use Result 2 to perform model selection. Specifically, we use our method to decide which of two given microscopic models is more likely to be the true model underlying some given data set. To this end, we use the Bayesian information criterion (BIC)^37^. The BIC for a model is the negative log-likelihood penalized by a term depending on the number of inferred parameters and number of measurement points. The model with the lower BIC is then chosen to be the true model.

As an example, we modified the SIRS model of equation (10) by including the possibility of a spontaneous, spatially homogeneous infection of susceptible agents, according to the reaction


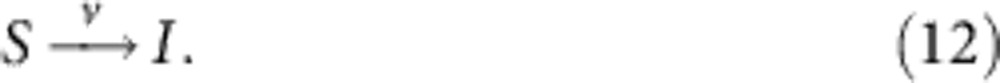


**Figure 5:**
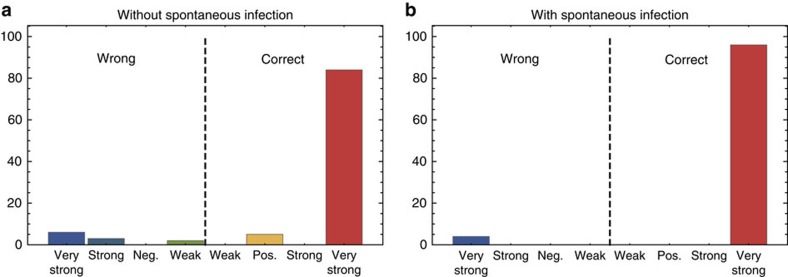
Model selection results for SIRS system. We use the Bayesian information criterion (BIC) for model selection for the SIRS system, with reactions in equation (10) and the additional spontaneous infection reaction in equation (12). The sign of the difference in the BIC numbers of the two models determines if the correct model is selected, and the corresponding magnitude how confident this choice is. We simulated 20 experiments for 5 parameter sets each. The figures show the combined results of these 100 experiments. (**a**) The true system used to generate the data does not include spontaneous infection. The parameter sets are the same as in Table 3. (**b**) The true system used to generate the data does include spontaneous infection. The parameter sets are the same as in Table 3, but with modified bimolecular infection rate that we set to *k*=10, 5, 10, 100 and 10, respectively. The spontaneous infection rate is set to *v*=0.002 for all parameter sets. In both cases, we observe that our method selects the correct model in >80% of the cases, and in most of these cases with ‘very strong' confidence. This demonstrates the strong performance of our method for this model selection problem.

We consider two scenarios: the true microscopic model used to generate the data does or does not contain the spontaneous infection reaction. In either case, we use our method to select the true model. To this end, we optimize the likelihood with respect to the parameters using our method for both models, and compare the corresponding BICs. Figure 5a,b shows the results for the two scenarios of true model without and with spontaneous infection, respectively. The figures show how often our method selected the right or wrong model, and with which confidence level. Each of the figures shows the combined results for 5 different parameter sets and 20 independent simulations for each parameter set. We find that our method chose the correct model in the vast majority of the cases (89% where the true system does not include spontaneous infection and 96% where the true system does contain spontaneous infection). Moreover, our method chooses the correct model with a ‘very strong' confidence in most of the cases. This shows that our method is well suited for the problem of model selection. The effectiveness of our model selection approach is remarkable, since the two mechanisms (spontaneous infection and contact infection) can lead to identifiability problems. Such problems are particularly acute when spatial heterogeneities even out rapidly, as in the case of fast diffusion: the few mistakes that our model selection approach makes are primarily due to random samples of the SRDP when the infection spreads particularly fast, so that, for most time points, the process is effectively equilibrated.

## Discussion

We considered two popular classes of models for studying stochasticity in spatio-temporal systems; SRDPs and spatio-temporal point processes. The two classes of models are both commonly used in many disciplines, such as epidemiology^14^,^38^ and social sciences^39^; however, they are widely perceived as conceptually distinct. SRDPs are microscopic, mechanistic descriptions used to predict the dynamics of spatially interacting particles, whereas point processes are typically used empirically to perform inference tasks for systems for which no microscopic description exists. The two approaches therefore seem to be orthogonal to each other.

However, in this paper, we have shown that the two methods are intimately related. By using the PR we established a Cox process representation of SRDPs, which is exact for certain classes of systems and approximate for others. This novel representation enables us to apply a wide range of statistical inference methods to SRDPs, which has not been possible so far. We applied the developed method to several example systems from systems biology and epidemiology, and obtained remarkably accurate results.

Since our method agrees with a deterministic rate equation description on the mean level, bimolecular reactions may lead to deviations from the true mean, which is known to be the case in some non-spatial scenarios^40^. Since in our method distributions are given as real Poisson mixtures, sub-Poissonian fluctuations cannot be captured. However, Gardiner showed that fluctuations of SRDPs are dominated by diffusion on small length scales and therefore Poissonian^33^, which may explain the accuracy of our method.

Most inference methods in the literature for SRDPs are either based on Brownian dynamics simulations or stochastic simulations of spatially discretized systems, using the RDME. Both approaches are computationally extremely expensive, and quickly become unfeasible for larger systems and in particular for inference purposes. In contrast, our method relies on the solution of (S)PDEs for which a rich literature of efficient numerical methods exists. For the studied example systems, our method turned out to be highly efficient: the computational time for inferring four unknown parameters for the SIRS system, for example, was found to be of the order of 10 s on a 3.1 GHz processor. We therefore expect our method to be applicable to significantly larger systems, containing more species and unknown parameters. Remarkably, simulating a single realization of the SIRS system from Brownian dynamics simulations took about an order of magnitude longer than the whole inference procedure, using our method, that is, optimizing the likelihood with respect to the parameters, indicating the immense computational costs of inference methods based on such simulations.

Having access to a likelihood function also provides a major advantage in handling model uncertainty: our results on a spatial SIRS model show that likelihood-based criteria can efficiently and accurately discriminate between competing models. This success raises the possibility that our approach could lay the foundations for structure learning of spatio-temporal stochastic systems: leveraging spatially resolved data not only to identify parameters, but to learn directly the mechanisms underlying the data. The availability of a likelihood approximation makes it in principle possible to borrow techniques from fields, where structure learning is more established and where efficient network learning algorithms based on regularized regression or random forests are routinely used, such as learning gene regulatory networks^41^,^42^.

Our approach can also readily handle spatial heterogeneity in the reaction or diffusion rates: the gene regulation example showed that the method can precisely identify simple geometric features of the system, such as the radius of the cell nucleus. While our examples are primarily illustrative of the methodology, and hence simple, it would be in principle straightforward to generalize the approach to SRDPs defined on complex geometries, such as the intracellular landscapes revealed by X-ray tomography^43^. Learning complex geometries directly from the data would potentially be more challenging, however, as it would generally require learning a large number of parameters.

While we believe that the derived representation of SRDPs in terms of Cox process brings clear advantages from a statistical point of view, it is also important to acknowledge the limitations implied by the employed mean-field approximations. Perhaps, the most important step in our approximation is the mean-field treatment of multi-time joint distributions in Result 2. As noticed before, this replaces direct dependencies between particle locations at different time points, with indirect dependences through the intensity field. This implies that self-excitatory behaviours, such as clustering, cannot be accurately captured; at best, these will be mimicked by a local increase in the intensity field within a Cox process framework. More complex point processes that can account for self-excitatory behaviour do exist^44^; in our opinion, it is a question of considerable theoretical interest whether such processes can also arise from a dynamical SRDP representation.

## Methods

### The chemical master equation

Consider a system of *N* species *X*_*i*_, *i*=1,...,*N* that interact stochastically via *R* reaction channels


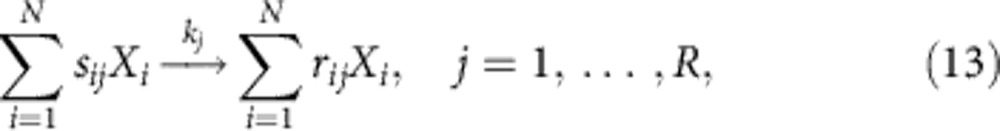


where *k*_*j*_ is the rate constant of the *j*th reaction, and the *s*_*ij*_ and *r*_*ij*_ are non-negative integer numbers. Define the stoichiometric matrix **S** as *S*_*ij*_=*r*_*ij*_−*s*_*ij*_; the *j*th reaction is of order *m* if 
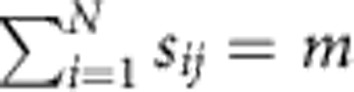
. We only consider reactions satisfying 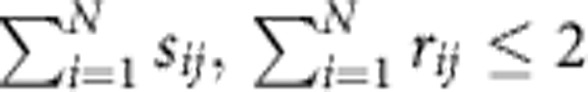, that is, reactions with a maximum of two reactant and a maximum of two product particles, since higher-order reactions rarely occur in nature. Denote as **n**=(*n*_1_,…,*n*_*N*_) the state of the system, where *n*_*i*_ is the copy number of species *X*_*i*_. Under well-mixed and dilute conditions, the time evolution of the (single time) marginal probability distribution of the system obeys the chemical master equation^30^


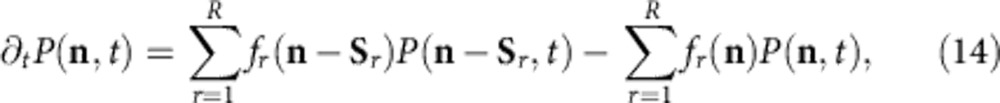


where **S**_*r*_ is the *r*th column of the stoichiometric matrix **S**. The propensity function *f*_*r*_(**n**)*dt* gives the probability for the *r*th reaction to happen in an infinitesimal time interval *dt* and is given by


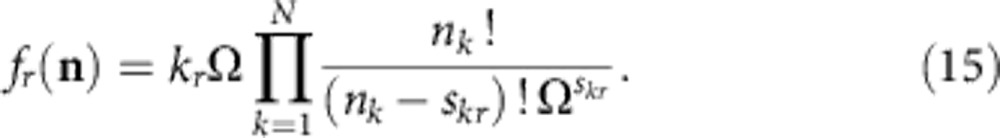


Here, Ω is the volume of the system.

### The Poisson representation

The PR makes the ansatz to write *P*(**n**,*t*) as a Poisson mixture^27^


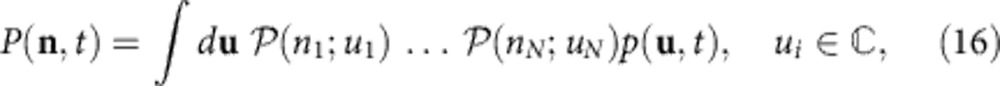


where **u**=(*u*_1_,…,*u*_*N*_) and 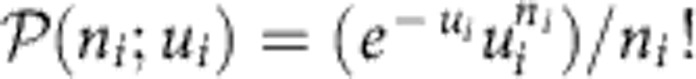 is a Poisson distribution in *n*_*i*_ with mean *u*_*i*_, and the *u*_*i*_ are complex in general. The integrals in equation (16) in general run over the whole complex plane for each *u*_*i*_. Using the ansatz (equation 16) in the generating function equation that can be derived from equation (14), one can derive the following PDE for the distribution *p*(**u**,*t*)^27^,


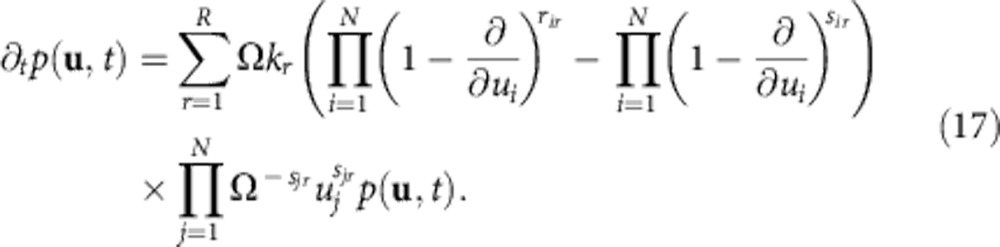


Note that this PDE generally involves derivatives of higher order than two, which means that *p*(**u**,*t*) can generally become negative, in which case it does not admit a probabilistic interpretation. However, since we only consider reactions satisfying 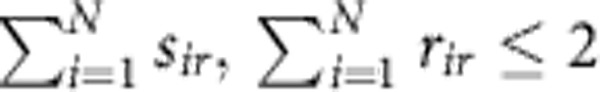, equation (17) simplifies to


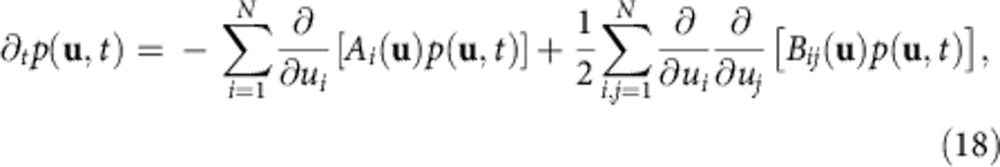


which is a Fokker–Planck equation with drift vector **A**(**u**) and diffusion matrix **B**(**u**) given by


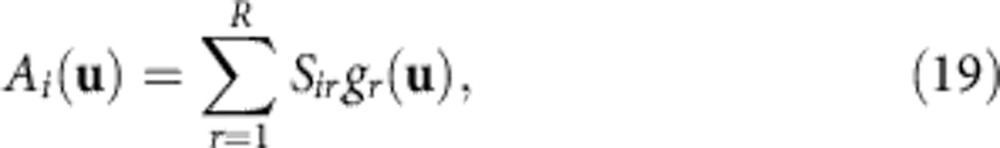



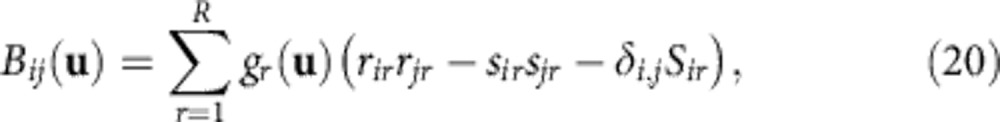



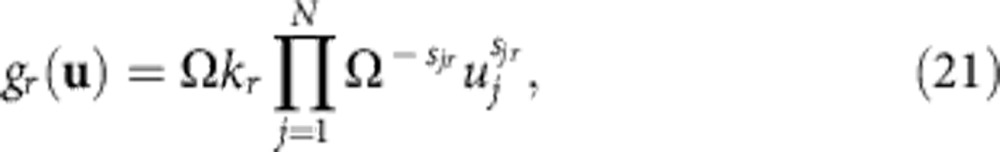


where *δ*_*i*,*j*_ denotes the Kronecker delta. The corresponding Langevin equation reads


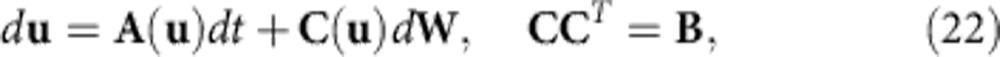


where **W** is a *l*-dimensional Wiener process and *l* is the number of columns of **C**.

Depending on the reactions in the system, the diffusion matrix may be zero, in which case the Langevin equation (equation 22) reduces to deterministic ordinary differential equations. On the other hand, depending on the reactions, **B**(**u**) is not positive semidefinite and thus **CC**^*T*^=**B** cannot be fulfilled for real **C**, which means that equation (18) is not a proper Fokker–Planck equation in real variables. Rather, it needs to be extended to complex space. Specifically, this is the case whenever the system contains bimolecular reactions or reactions with two non-identical product molecules.

An important property of the PR is that the mean values of the particle numbers *n*_*i*_ are equal to the mean values of the corresponding PR variables *u*_*i*_, that is, 〈*n*_*i*_〉=〈*u*_*i*_〉.

### The reaction–diffusion master equation

Consider a system as in equation (13), but in an *M*-dimensional volume discretized into *L* cubic compartments of edge length *h* and volume *h*^*M*^. Denote as **n**=(*n*_1_^1^,…,*n*_*N*_^1^,…,*n*_1_^*L*^,…,*n*_*N*_^*L*^) the state of the system, where 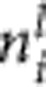 is the copy number of species *X*_*i*_ in the *l*th compartment. Under well-mixed and dilute conditions in each compartment, the reaction dynamics in each compartment is governed by a corresponding chemical master equation as in equation (14). If we model diffusion of species *X*_*i*_ between neighbouring compartments by linear reactions with rate constant *d*_*i*_=*D*_*i*_/*h*^2^, where *D*_*i*_ is the microscopic diffusion constant of species *X*_*i*_, the time evolution of the (single time) marginal probability distribution of the system obeys the RDME^30^:


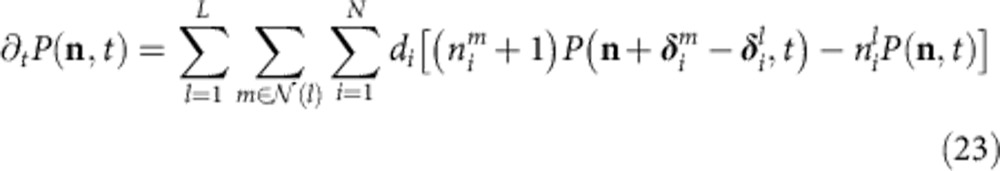



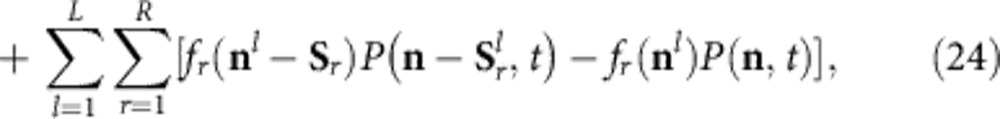


where *f*_*r*_(**n**^*l*^) is the propensity function of the *r*th reaction evaluated at the state vector 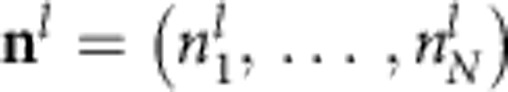 of the *l*th compartment, 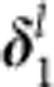 is a vector of length *N* × *L* with the entry corresponding to species *X*_*i*_ in the *l*th compartment equal to 1 and all other entries zero, and 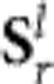 is a vector of length *N* × *L* with the entries corresponding to the *l*th compartment equal to the *r*th row of the stoichiometric matrix **S** and zero otherwise.

### Real-valued Poisson representation in space

We next apply the PR to the RDME in equations (23) and (24) after applying the mean-field approximations defined in the Results section to bimolecular reactions and reactions with two non-identical product molecules, and subsequently take the continuum limit. Consider first the diffusion term in equation (23). Since different species do not interact with each other if there are no chemical reactions happening, we can consider a system containing only a single species, say species *X*_1_, for which equation (23) reduces to


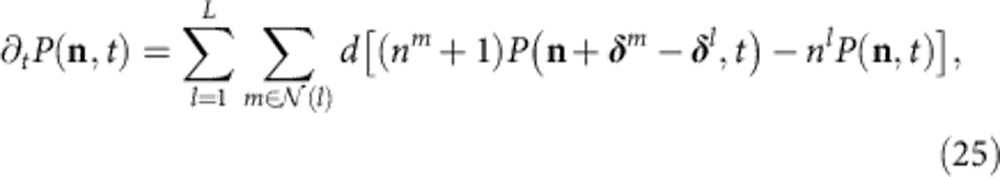


where **n**=(*n*^1^,…,*n*^*L*^), *n*^*m*^ is the number of *X*_1_ particles in the *m*th compartment, ***δ***^*m*^ is a vector with a one in the *m*th entry and zero otherwise, *d* is the diffusion constant of species *X*_1_ and the sum over *m* runs over all neighbouring compartments 
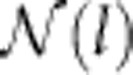
 of the *l*th compartment. For this system, the PR is real and deterministic, and we use the PR without any approximations. The corresponding Langevin equation reads


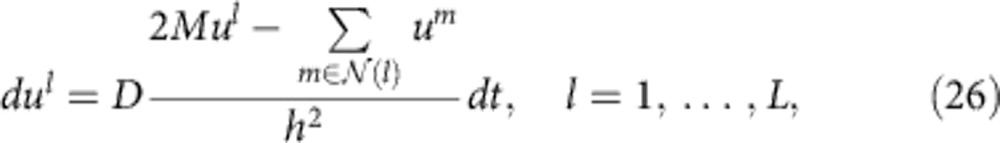


where *M* is the spatial dimension of the system and *D*=*dh*^2^ the microscopic diffusion constant. Since the sum over *m* runs over all adjacent compartments of the *l*th compartment, the fraction in equation (26) is just the discretized version of the Laplace operator 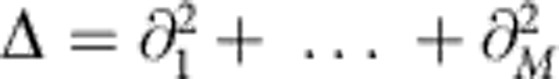. Introducing a discretized density field *u*(*x*^*l*^)=*u*^*l*^/*h*^*M*^, where *x*^*l*^ is the centre of the *l*th compartment, and taking the continuum limit of equation (26), we get the PDE


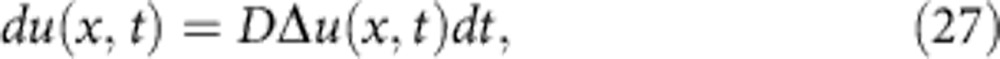


which is just the diffusion equation for the field *u*(*x*,*t*).

Consider next the reaction term of the RDME given in equation (24). Since reactions only occur within compartments, we can treat the compartments independently of each other. For a single compartment, equation (24) then reduces to the chemical master equation given in equation (14). Here, however, we first apply the approximations defined in the Results section to bimolecular reactions and reactions with two non-identical product molecules (see Supplementary Methods for more details). These approximations lead to a real-valued PR and only reactions with two identical product molecules lead to stochastic terms. The PR Langevin equation hence simplifies to


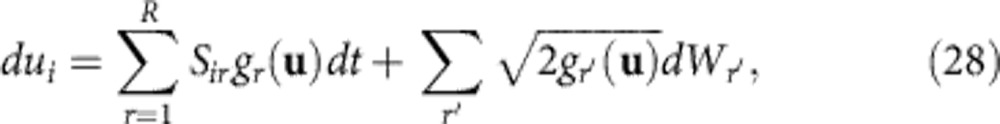


where **u**=(*u*_1_,…,*u*_*N*_) and the sum over *r*′ runs over all reactions with two product particles of species *X*_*i*_. The propensities *g*_*r*_(**u**) are obtained by replacing the *n*_*i*_ variables with *u*_*i*_ variables and Ω with *h*^*M*^ in the expressions for the *f*_*r*_ propensities of the approximated reactions. The factor of two in the square root in equation (28) comes from the fact that two identical molecules become produced in these reactions. Reintroducing the label *l* denoting the compartment number in equation (28), and the species label *i* in equation (27), we can add the two contributions to obtain


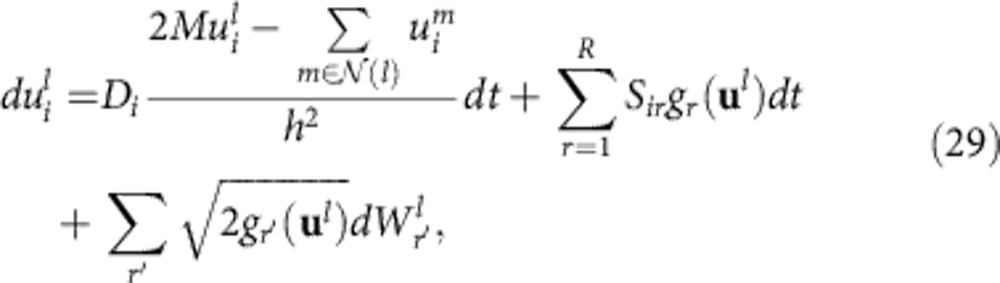


where 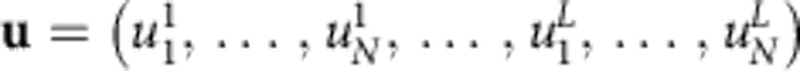 and *u*_*i*_^*l*^ is the PR variable of species *X*_*i*_ in the *l*th compartment. If we again define discretized density fields 
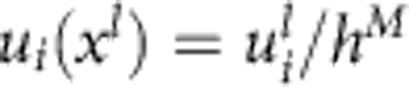
, where *x*^*l*^ is the centre of the *l*th compartment, and 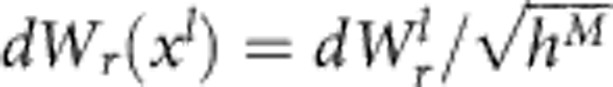, we can take the continuum limit of equation (29) that leads to the real-valued SPDE for the intensity fields given in equation (4). The *g*_*r*_(**u**(*x*,*t*)) therein are not functions of single PR variables anymore, but rather functionals of the space-dependent intensity field vector **u**(*x*,*t*)=(*u*_1_(*x*,*t*),…,*u*_*N*_(*x*,*t*)). They are obtained by taking the corresponding propensity functions *f*_*r*_(**n**) of the approximate reactions in real space, replacing *n*_*i*_→*u*_*i*_(*x*,*t*) and 〈*n*_*i*_〉→〈*u*_*i*_(*x*,*t*)〉, and omitting Ω factors. The latter can be identified with *h*^*M*^ here, and hence get absorbed in the definition of the intensity fields given below equation (29).

As an example, consider the reaction *A*+*B*→∅. The non-spatial propensity in real space for this reaction is *f*(*n*_*A*_,*n*_*B*_)=*kn*_*A*_*n*_*B*_/Ω. However, since this is a bimolecular reaction and hence leads to a complex-valued PR, we replace it by two reactions *A*→∅ and *B*→∅, with propensities *f*_1_(*n*_*A*_,*n*_*B*_)=*k*〈*n*_*B*_〉*n*_*A*_/Ω and *f*_2_(*n*_*A*_,*n*_*B*_)=*k*〈*n*_*A*_〉*n*_*B*_/Ω, respectively. By replacing *n*_*i*_→*u*_*i*_(*x*,*t*) and 〈*n*_*i*_〉→〈*u*_*i*_(*x*,*t*)〉, and omitting Ω terms, we thus obtain the corresponding propensity functions in spatial PR space as *g*_1_(*u*_*A*_(*x*,*t*),*u*_*B*_(*x*,*t*))=*k*〈*u*_*B*_(*x*,*t*)〉*u*_*A*_(*x*,*t*) and *g*_2_(*u*_*A*_(*x*,*t*),*u*_*B*_(*x*,*t*))=*k*〈*u*_*A*_(*x*,*t*)〉*u*_*B*_(*x*,*t*), respectively. See Supplementary Methods for more details and examples.

### Poisson and Cox processes

A (spatial) Poisson process on a spatial region 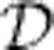 of arbitrary dimension defines a measure on countable unions of zero-dimensional subsets (points) of 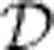. A Poisson process is often characterized by an intensity function 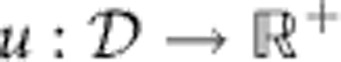, giving the probability density of finding a point in an infinitesimal region around *x*. Now, let *N*(*A*) denote the number of points in a subregion 
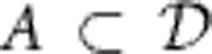
. Then *N*(*A*) is a Poisson random variable with mean given by the integral of *u*(·) over *A*:


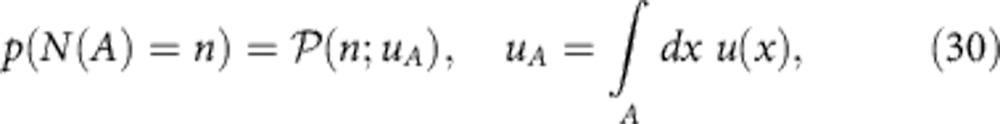


where 
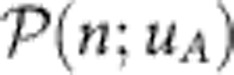
 is a Poisson distribution in *n* with mean *u*_*A*_.

A (spatial) Cox process is a generalization of a Poisson process and also called ‘doubly stochastic process', in the sense that the intensity function *u* is itself a random process. Conditioned on the intensity *u*, the Cox process reduces to a Poisson process. The distribution of the number of points in a subregion 
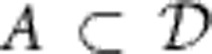
 is hence a mixture of Poisson distributions,


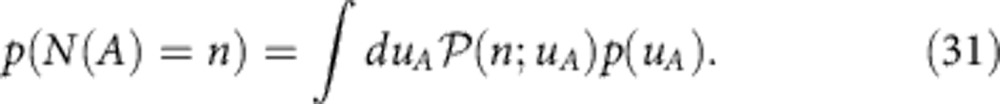


Since we are interested in dynamical systems, we will assume time-dependent intensities 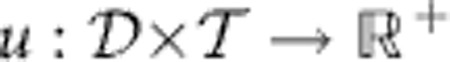, where 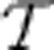 is a finite real interval denoting time. We then require that for any fixed time point 
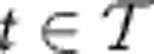
, the process is a spatial Poisson (Cox) process with intensity *u*(·,*t*). In the case of a Poisson (Cox) process, the intensity *u* may, for example, be defined as the solution of a PDE (SPDE).

### Code availability

The software used for the studied examples is available as Supplementary Data 1–4.

### Data availability

The *Drosophila* Bicoid data used in this study is available from the FlyEx database, http://urchin.spbcas.ru/flyex/.

## Additional information

**How to cite this article:** Schnoerr, D. *et al*. Cox process representation and inference for stochastic reaction–diffusion processes. *Nat. Commun.* 7:11729 doi: 10.1038/ncomms11729 (2016).

## Supplementary Material

Supplementary Information Supplementary Methods, Supplementary References

Supplementary Dataset 1 Matlab code performing parameter inference for one simulated data set for the gene expression system.

Supplementary Dataset 2 Matlab code performing parameter inference for one simulated data set for the gene expression system with additional autocatalytic reaction.

Supplementary Dataset 3 Matlab code performing parameter inference for one simulated data set for the SIRS system.

Supplementary Dataset 4 Matlab code performing parameter inference for one embryo.

## Figures and Tables

**Table 1:** Inference results for gene expression system.

	***r***	***d***_**m**_	***d***_**p**_	***m***_**1**_	***m***_**2**_	***p***_**1**_	***p***_**2**_
Exact	0.3	0.1	0.1	20	0.5	20	0.2
Inferred	0.31 (0.06)	0.12 (0.08)	0.14 (0.06)	23 (12)	0.51 (0.4)	26 (18)	0.25 (0.1)

The table shows the inferred parameter values for the gene expression system illustrated in Fig. 2, with reactions in equations (7) and (8). We assume the measurements of the protein, while the mRNA is unobserved. The inference is carried out by maximizing the likelihood of simulated data for 30 measurement points separated by Δ*t*=0.5. This procedure is carried out for 100 simulated data sets and the mean value and s.d. (in parenthesis) of the inferred results are displayed.

**Table 2:** Inference results for gene expression system with additional autocatalytic reaction.

	***r***	***d***_**m**_	***d***_**p**_	***m***_**1**_	***m***_**2**_	***p***_**1**_	***p***_**2**_
Exact	0.3	0.1	0.1	20	0.5	20	0.2
Inferred	0.30 (0.05)	0.14 (0.08)	0.088 (0.03)	27 (17)	0.57 (0.3)	24 (21)	0.19 (0.08)

The table shows the inferred parameter values for the gene expression system illustrated in Fig. 2, with reactions in equations (7) and (8), and the additional autocatalytic reaction in equation (9). Since, only the difference *p*_2_−*p*_3_ is identifiable, we fix *p*_3_=0.01 and infer the other seven parameters. The table shows the average and s.d. (in parenthesis) of the inference results for 100 simulated data set.

**Table 3:** Inference results for SIRS system.

	**10**^**3**^**× d**	**10 × r**	**10 × s**	**10**^**3**^**×*****k***^**PR**^	**k**	**w**	***S***_**ini**_
Exact	1	0.2	2	—	100	0.01	200
Inferred	0.8 (0.3)	0.19 (0.09)	1.8 (1.2)	2.5 (0.5)	—	—	
Exact	1	0.2	2	—	100	0.01	300
Inferred	0.9 (0.4)	0.15 (0.06)	1.4 (0.9)	2.4 (0.5)	—	—	
Exact	1	2	2	—	100	0.02	200
Inferred	1.0 (0.6)	1.6 (0.7)	1.5 (1.0)	3.4 (1.1)	—	—	
Exact	1	0.2	2	—	1000	0.005	200
Inferred	0.8 (0.4)	0.21 (0.11)	2.2 (1.6)	2.4 (0.5)	—	—	
Exact	2	0.2	2	—	100	0.01	100
Inferred	1.6 (0.8)	0.19 (0.09)	1.9 (1.2)	4.6 (1.1)	—	—	

The table shows the results for parameter inference for the SIRS system, with reactions given in equation (10). The inference is carried out by maximizing the likelihood of the simulated data for 40 measurement points. This procedure is carried out for 200 simulated data sets, and the mean value and s.d. (in parenthesis) of the inferred results are displayed.

## References

[b1] Bullara D. & De Decker Y. Pigment cell movement is not required for generation of Turing patterns in zebrafish skin. Nat. Commun. 6, 6971 (2015). 25959141 10.1038/ncomms7971 PMC4432648

[b2] Metzler R. The future is noisy: the role of spatial fluctuations in genetic switching. Phys. Rev. Lett. 87, 068103 (2001). 11497866 10.1103/PhysRevLett.87.068103

[b3] Elf J. & Ehrenberg M. Spontaneous separation of bi-stable biochemical systems into spatial domains of opposite phases. Syst. Biol. 1, 230–236 (2004). 10.1049/sb:20045021 17051695

[b4] Takahashi K., Tanase-Nicola S. & Ten Wolde P. R. Spatio-temporal correlations can drastically change the response of a MAPK pathway. Proc. Natl Acad. Sci. USA 107, 2473–2478 (2010). 20133748 10.1073/pnas.0906885107 PMC2811204

[b5] Short M. B., Brantingham P. J., Bertozzi A. L. & Tita G. E. Dissipation and displacement of hotspots in reaction-diffusion models of crime. Proc. Natl Acad. Sci. USA 107, 3961–3965 (2010). 20176972 10.1073/pnas.0910921107 PMC2840109

[b6] Mahmutovic A., Fange D., Berg O. G. & Elf J. Lost in presumption: stochastic reactions in spatial models. Nat. Methods 9, 1163–1166 (2012). 23223170 10.1038/nmeth.2253

[b7] Yates C. A. . Inherent noise can facilitate coherence in collective swarm motion. Proc. Natl Acad. Sci. USA 106, 5464–5469 (2009). 19336580 10.1073/pnas.0811195106 PMC2667078

[b8] Cottrell D., Swain P. S. & Tupper P. F. Stochastic branching-diffusion models for gene expression. Proc. Natl Acad. Sci. USA 109, 9699–9704 (2012). 22660929 10.1073/pnas.1201103109 PMC3382520

[b9] Doi M. Second quantization representation for classical many-particle system. J. Phys. A 9, 1465 (1976).

[b10] Doi M. Stochastic theory of diffusion-controlled reaction. J. Phys. A 9, 1479 (1976).

[b11] Bicknell B. A., Dayan P. & Goodhill G. J. The limits of chemosensation vary across dimensions. Nat. Commun. 6, 7468 (2015). 26088726 10.1038/ncomms8468 PMC4557358

[b12] Grima R. & Schnell S. A systematic investigation of the rate laws valid in intracellular environments. Biophys. Chem. 124, 1–10 (2006). 16781049 10.1016/j.bpc.2006.04.019

[b13] Holmes G. R. . Repelled from the wound, or randomly dispersed? Reverse migration behaviour of neutrophils characterized by dynamic modelling. J. R. Soc. Interface 9, 3229–3239 (2012). 22951343 10.1098/rsif.2012.0542 PMC3481594

[b14] Davies T. P., Fry H. M., Wilson A. G. & Bishop S. R. A mathematical model of the London riots and their policing. Sci. Rep. 3, 1303 (2013). 23425781 10.1038/srep01303 PMC3578270

[b15] Gardiner C. W., McNeil K. J., Walls D. F. & Matheson I. S. Correlations in stochastic theories of chemical reactions. J. Stat. Phys. 14, 307–331 (1976).

[b16] Fange D., Berg O. G., Sjöberg P. & Elf J. Stochastic reaction-diffusion kinetics in the microscopic limit. Proc. Natl Acad. Sci. USA 107, 19820–19825 (2010). 21041672 10.1073/pnas.1006565107 PMC2993376

[b17] Isaacson S. A. Relationship between the reaction-diffusion master equation and particle tracking models. J. Phys. A 41, 065003 (2008).

[b18] van Zon J. S. & Ten Wolde P. R. Simulating biochemical networks at the particle level and in time and space: Green's function reaction dynamics. Phys. Rev. Lett. 94, 128103 (2005). 15903966 10.1103/PhysRevLett.94.128103

[b19] Isaacson S. A. & Peskin C. S. Incorporating diffusion in complex geometries into stochastic chemical kinetics simulations. SIAM J. Sci. Comput. 28, 47–74 (2006).

[b20] Erban R. & Chapman S. J. Stochastic modelling of reaction-diffusion processes: algorithms for bimolecular reactions. Phys. Biol. 6, 046001 (2009). 19700812 10.1088/1478-3975/6/4/046001

[b21] Drawert B., Lawson M. J., Petzold L. & Khammash M. The diffusive finite state projection algorithm for efficient simulation of the stochastic reaction-diffusion master equation. J. Chem. Phys. 132, 074101 (2010). 20170209 10.1063/1.3310809 PMC2905448

[b22] Ferm L., Hellander A. & Lötstedt P. An adaptive algorithm for simulation of stochastic reaction-diffusion processes. J. Comput. Phys. 229, 343–360 (2010).

[b23] Franz B., Flegg M. B., Chapman S. J. & Erban R. Multiscale reaction-diffusion algorithms: PDE-assisted Brownian dynamics. SIAM J. Appl. Math. 73, 1224–1247 (2013).

[b24] Fu J., Wu S., Li H. & Petzold L. R. The time dependent propensity function for acceleration of spatial stochastic simulation of reaction-diffusion systems. J. Comput. Phys. 274, 524–549 (2014). 26609185 10.1016/j.jcp.2014.06.025 PMC4655327

[b25] Dewar M. A., Kadirkamanathan V., Opper M. & Sanguinetti G. Parameter estimation and inference for stochastic reaction-diffusion systems: application to morphogenesis in D. melanogaster. BMC Syst. Biol. 4, 21 (2010). 20219114 10.1186/1752-0509-4-21 PMC2848629

[b26] Ruttor A. & Opper M. Approximate parameter inference in a stochastic reaction-diffusion model. AISTATS 9, 669–676 (2010).

[b27] Gardiner C. W. & Chaturvedi S. The Poisson representation. I. A new technique for chemical master equations. J. Stat. Phys. 17, 429–468 (1977).

[b28] Cressie N. A. C. & Wikle C. K. Statistics for Spatio-temporal Data Wiley (2011).

[b29] Cseke B., Zammit Mangion A., Heskes T. & Sanguinetti G. Sparse approximate inference for spatio-temporal point process models. JASA (2015) (in the press).

[b30] Gillespie D. T., Hellander A. & Petzold L. R. Perspective: stochastic algorithms for chemical kinetics. J. Chem. Phys. 138, 170901 (2013). 23656106 10.1063/1.4801941 PMC3656953

[b31] Thomas P., Straube A. V. & Grima R. Stochastic theory of large-scale enzyme-reaction networks: Finite copy number corrections to rate equation models. J. Chem. Phys. 133, 195101 (2010). 21090871 10.1063/1.3505552

[b32] Kingman J. F. C. Poisson Processes Oxford Univ. Press (1992).

[b33] Gardiner C. W. Handbook of Stochastic Methods Springer (1985).

[b34] Peruani F. & Lee C. F. Fluctuations and the role of collision duration in reaction-diffusion systems. EPL 102, 58001 (2013).

[b35] Abdullah M., Cooper C. & Draief M. in Approximation, Randomization, and Combinatorial Optimization. Algorithms and Techniques: 14th International Workshop, APPROX 2011, and 15th International Workshop, RANDOM 2011 351–364Springer (2011).

[b36] Pisarev A., Poustelnikova E., Samsonova M. & Reinitz J. FlyEx, the quantitative atlas on segmentation gene expression at cellular resolution. Nucleic Acids Res. 37, D560–D566 (2009). 18953041 10.1093/nar/gkn717 PMC2686593

[b37] Schwarz G. E. Estimating the dimension of a model. Ann. Stat. 6, 461–464 (1978).

[b38] Grell K. . A three-dimensional point process model for the spatial distribution of disease occurrence in relation to an exposure source. Stat. Med. 34, 3170–3180 (2015). 26011698 10.1002/sim.6538

[b39] Zammit-Mangion A., Dewar M., Kadirkamanathan V. & Sanguinetti G. Point process modelling of the Afghan War Diary. Proc. Natl Acad. Sci. USA 109, 12414–12419 (2012). 22802667 10.1073/pnas.1203177109 PMC3411944

[b40] Ramaswamy R., Gonzalez-Segredo N., Sbalzarini I. F. & Grima R. Discreteness-induced concentration inversion in mesoscopic chemical systems. Nat. Commun. 3, 779 (2012). 22491327 10.1038/ncomms1775

[b41] Bonneau R. . The Inferelator: an algorithm for learning parsimonious regulatory networks from systems-biology data sets *de novo*. Genome Biol. 7, R36 (2006). 16686963 10.1186/gb-2006-7-5-r36 PMC1779511

[b42] Huynh-Thu V.-A. & Sanguinetti G. Combining tree-based and dynamical systems for the inference of gene regulatory networks. Bioinformatics 31, 1614–1622 (2015). 25573916 10.1093/bioinformatics/btu863 PMC4426834

[b43] Do M., Isaacson S. A., McDermott G., Le Gros M. A. & Larabell C. A. Imaging and Characterizing Cells using Tomography. Arch. Biochem. Biophys. 581, 111–121 (2015). 25602704 10.1016/j.abb.2015.01.011 PMC4506273

[b44] Hawkes A. G. & Oakes D. A cluster process representation of a self-exciting process. J. Appl. Prob. 11, 493–503 (1974).

