# Supplementary material for: Cox process representation and inference for stochastic reaction–diffusion processes
Source: Nat Commun. 2016 May 25;7:11729. doi: 10.1038/ncomms11729 (PMC4894951; doi:10.1038/ncomms11729)
Supplement: Supplementary Information — Supplementary Methods, Supplementary References [file ncomms11729-s1.pdf]

# Supplementary methods

## 1 Cox process representation and mean-field approximations

### 1.1 Classification of reactions w.r.t. to their Poisson representation

As mentioned in the Methods section, the Poisson representation (PR) becomes complex depending on the reactions in the system. Supplementary Table 1 shows a classification of different types of elementary reactions in terms of the behaviour of the corresponding diffusion matrix $\mathbf{B}(\mathbf{u})$. We note that this strict classification of course only holds if the considered reaction is the only reaction in the system. If there are several reactions happening, the system typically behaves as the entry in Supplementary Table 1 corresponding to the reaction of highest type.

The behaviours of the PR are quite intuitive: for reactions of Type I, it is well-known that fluctuations are Poissonian, which manifests itself in a deterministic PR. Note that if the Poisson representation is real-valued, the probability distribution of the molecule numbers in the PR ansatz given in Equation (3) in the main text is a real-valued mixture of Poisson distributions, for which it is well-known that the resulting distributions are super-Poissonian. Reactions of Type II, for which fluctuations are super-Poissonian, therefore have real and stochastic PRs. It is easy to see that reactions of Type III and IV, however, cannot be represented in this way: a zeroth or first order reaction with two *non-identical* product molecules, i.e., of Type III, imposes a constraint on the particle numbers. For the reaction $\varnothing \to A + B$ for example, the particle numbers of species $A$ and $B$ differ by a constant integer number (depending on the initial condition). Conditioned on the molecule number of $A$, $B$ is a delta distribution, which clearly cannot be achieved by a real Poisson mixture, and the PR has to be complex. Bimolecular reactions give rise to similar constraints or may lead to sub-Poissonian fluctuations, and hence their PR has to be complex-valued. We therefore approximate reactions of Type III and Type IV as described in the following.

### 1.2 Approximation of Type III and IV reactions

We would like a real PR for general reaction networks. We therefore have to approximate reactions of Type III and IV. Consider first reactions of Type IV, where two molecules react with each other. We approximate this type of reactions in a *mean-field* type of sense: we replace the interaction of the two molecules with each other by two unimolecular reactions whose propensity functions depend on the particle number of one species and the *mean value* of the respective other species. For instance, the reaction

$$A + B \xrightarrow{k} \varnothing, \quad f(\mathbf{n}) = \frac{k}{\Omega} n_B n_A, \tag{1}$$

becomes replaced by the two reactions

$$A \xrightarrow{k\langle n_B\rangle/\Omega} \varnothing, \quad f(\mathbf{n}) = \frac{k}{\Omega}\langle n_B\rangle n_A, \tag{2}$$

$$B \xrightarrow{k\langle n_A\rangle/\Omega} \varnothing, \quad f(\mathbf{n}) = \frac{k}{\Omega}\langle n_A\rangle n_B, \tag{3}$$

where $\langle n_A\rangle$ and $\langle n_B\rangle$ denote the mean values of the molecule numbers of species $A$ and $B$, respectively, and $\Omega$ is the volume of the system. The reactions (2) and (3) thus correspond to linear reactions with one reactant and zero product molecules. The corresponding PR is therefore real and deterministic. Since the mean values of the corresponding PR variables, say $u_A$ and $u_B$, are equal to the means of $\langle n_A\rangle$ or $\langle n_B\rangle$, the rate constants in PR space simply become rescaled by

Supplementary Table 1: Classification of different types of reactions w.r.t. to their Poisson representation. If different types of reactions are happening, the PR typically behave like the reaction of highest type.

| reaction types | | | | PR |
|---|---|---|---|---|
| Type | stoichiometry | description | examples | |
| I | $\sum_i s_{ir} \leq 1$ <br> $\sum_i r_{ir} \leq 1$ | zero or one reactant and product molecules | $\varnothing \to A$ <br> $A \to \varnothing$ <br> $A \to B$ | real and determ. |
| II | $\sum_i s_{ir} \leq 1$ <br> $r_{ir} = 2$ for one $i$ and zero otherwise | zero or one reactant; two *identical* product molecules | $\varnothing \to A + A$ <br> $A \to A + A$ <br> $B \to A + A$ | real and stoch. |
| III | $\sum_i s_{ir} \leq 1$ <br> $r_{ir} = r_{jr} = 1$ for two $i \neq j$ and zero otherwise | zero or one reactant; two *non-identical* product molecules | $\varnothing \to A + B$ <br> $A \to A + B$ <br> $A \to B + C$ | complex and stoch. |
| IV | $\sum_i s_{ir} = 2$ <br> $\sum_i r_{ir} \leq 2$ | two reactant molecules | $A + A \to \dots$ <br> $A + B \to \dots$ | complex and stoch. |

$\langle u_A \rangle/\Omega$ and $\langle u_B \rangle/\Omega$, respectively. Specifically, if there are no other reactions happening in the system, the PR Langevin equations read

$$du_A = -\frac{k}{\Omega}\langle u_B \rangle u_A dt, \tag{4}$$

$$du_B = -\frac{k}{\Omega}\langle u_A \rangle u_B dt. \tag{5}$$

Consider now a bimolecular reaction with two identical reactant molecules,

$$A + A \xrightarrow{k} \varnothing, \quad f(\mathbf{n}) = \frac{k}{\Omega} n_A(n_A - 1). \tag{6}$$

For such reactions, we replace the interaction of $A$ with itself by the interaction of $A$ with its mean,

$$A \xrightarrow{k\langle n_A \rangle/\Omega} \varnothing, \quad f(\mathbf{n}) = \frac{k}{\Omega}\langle n_A \rangle n_A. \tag{7}$$

In PR space, this leads to the Langevin equation for $A$,

$$du_A = -\frac{k}{\Omega}\langle u_A \rangle u_A dt. \tag{8}$$

Consider next the following reaction of Type III (c.f. Supplementary Table 1)

$$A \xrightarrow{k} A + B, \quad f(\mathbf{n}) = k n_A, \tag{9}$$

which can be approximated in a similar fashion as the bimolecular reactions before: we replace the dependence of the creation of $B$ molecules on $A$ molecules by a dependence on the mean of the later, i.e. $\langle n_A \rangle$,

$$\varnothing \xrightarrow{k\langle n_A \rangle} B, \quad f(\mathbf{n}) = k\langle n_A \rangle. \tag{10}$$

Supplementary Table 2: Reactions of Types III and IV and their approximate reactions. The corresponding propensities in PR space for the approximate system are obtained by replacing $n_A$ and $n_B$ with $u_A$ and $u_B$, respectively. $n_A$ and $n_B$ denote the particle number variables of species $A$ and $B$, respectively, and $u_A$ and $u_B$ the corresponding PR variables.

| reactions | | approximation | |
|---|---|---|---|
| type | propensity | type | propensity |
| $A + B \to \dots$ | $kn_A n_B/\Omega$ | $A \to \dots$<br>$B \to \dots$ | $k\langle n_B\rangle n_A/\Omega$<br>$k\langle n_A\rangle n_B/\Omega$ |
| $A + A \to \dots$ | $kn_A(n_A - 1)/\Omega$ | $A \to \dots$ | $k\langle n_A\rangle n_A/\Omega$ |
| $A \to A + B$ | $kn_A$ | $\varnothing \to B$ | $k\langle n_A\rangle$ |
| $\varnothing \to B + C$ | $\Omega k$ | $\varnothing \to B$<br>$\varnothing \to C$ | $\Omega k$<br>$\Omega k$ |
| $A \to B + C$ | $kn_A$ | $A \to B$<br>$A \to C$ | $kn_A$<br>$kn_A$ |

For the other two Type III reactions,

$$\varnothing \xrightarrow{k} B + C, \quad f(\mathbf{n}) = k\Omega, \tag{11}$$

$$A \xrightarrow{k} B + C, \quad f(\mathbf{n}) = kn_A, \tag{12}$$

we have to decouple the productions of $B$ and $C$, which can be achieved by approximating the reactions by

$$\varnothing \xrightarrow{k} B, \quad \varnothing \xrightarrow{k} C, \quad f(\mathbf{n}) = k\Omega, \tag{13}$$

$$A \xrightarrow{k} B, \quad A \xrightarrow{k} C, \quad f(\mathbf{n}) = kn_A. \tag{14}$$

While (11) correlates the molecule numbers of species $B$ and $C$, we have effectively decorrelated $B$ and $C$ by introducing the reactions (13) and (13).

Supplementary Table 2 summarises the approximations for all reactions of Type III and IV. Note that bimolecular reactions (Type IV) with two identical product molecules under these approximations still lead to stochastic PRs. Note also that depending on the reaction, a combination of the used approximations has to be performed, for example for the reactions $A + B \to A + C$ or $A + A \to C + D$.

**Example**

As an example, consider the following reaction system

$$X \xrightarrow{k_1} X + X, \quad X + X \xrightarrow{k_2} \varnothing. \tag{15}$$

The corresponding stoichiometric matrix reads

$$\mathbf{S} = (1, -2). \tag{16}$$

The first reaction in (15) is of Type II and thus does not need to be approximated. The corresponding non-spatial propensity function in real space is given by $f_1(n) = k_1 n$, where $n$ is the variable denoting the number of $X$ particles. The second reaction in Eq. (15) is of Type IV and hence needs to be approximated. According to Supplementary Table 2 we approximate it by the reaction $X \xrightarrow{k_2\langle n\rangle/\Omega} \varnothing$ with propensity $f_2(n) = k_2\langle n\rangle n/\Omega$. The corresponding propensity functions in spatial PR space are obtained by replacing $n \to u(x,t)$ and $\langle n\rangle \to \langle u(x,t)\rangle$, where

$u(x,t)$ is the PR field of species $X$. We thus have

$$\begin{aligned} X &\xrightarrow{k_1} X+X, \quad f_1(n)=k_1 n, \\ &\downarrow \\ X &\xrightarrow{k_1} X+X, \quad g_1(u(x,t))=k_1 u(x,t), \end{aligned} \tag{17}$$

for the first reaction and

$$\begin{aligned} X+X &\xrightarrow{k_2} \varnothing, \quad f_2(n)=\frac{k_2}{\Omega}n(n-1), \\ &\downarrow \\ X &\xrightarrow{k_2} \varnothing, \quad f_2(n)=\frac{k_2}{\Omega}\langle n\rangle n, \\ &\downarrow \\ X &\xrightarrow{k_2} \varnothing, \quad g_2(u(x,t))=k_2\langle u(x,t)\rangle u(x,t), \end{aligned} \tag{18}$$

for the second reaction. The corresponding stoichiometric matrix becomes

$$\mathbf{S}=(1,-1). \tag{19}$$

Using the general stochastic partial differential equation (SPDE )for intensity fields given in (4) in the main text we hence obtain the for the intensity field $u(x,t)$,

$$\begin{aligned} du(x,t) = [D\Delta u(x,t) + k_1 u(x,t) - k_2\langle u(x,t)\rangle u(x,t)]dt \\ + \sqrt{2k_2\langle u(x,t)\rangle u(x,t)}dW(x,t). \end{aligned} \tag{20}$$

We would like to emphasise that $\langle u(x,t)\rangle$ denotes the local expectation of the stochastic intensity field $u(x,t)$ and not a spatial averaging.

## 1.3 Proof of Remark 1 and Result 1

The proof of Remark 1 and Result 1 relies on the SPDE in Equation (4) in the main text and its derivation given in the Methods section. For simplicity, we consider a one-dimensional system with one species $X$ in the interval $[0,1]$ here. Consider the PR of the RDME for approximated reactions given in Equation (29). Consider first a system involving only reactions of Type I. In that case we do not have to perform any approximations to obtain Equation (29) and the second sum including the noise terms vanishes, i.e., Equation (29) reduces to a PDE. For deterministic initial conditions the $u_i$ thus remain deterministic, and the probability distribution of $n^l$ in compartment $l$ at time $t$ is given by a Poisson distribution with mean value $u^l(t)$. The mean number of molecules in an interval $I=[(m_1-\frac{1}{2})h,(m_2+\frac{1}{2})h], m_1<m_2\in\mathbb{N}$ at time $t$ is thus

$$\langle N(I,t)\rangle = \sum_{i=m_1}^{m_2}\langle n^i\rangle = \sum_{i=m_1}^{m_2}\langle u^i\rangle = \sum_{i=m_1}^{m_2} u^i, \tag{21}$$

where $N(I,t)=\sum_{i=m_1}^{m_2} n^i$. Since the $n_i$ are independent Poisson random variables, $N(I,t)$ is also a Poisson random variable with mean $\langle N(I,t)\rangle = \sum_{i=m_1}^{m_2} u^i(t)$.

Defining $u^i/h \to u(x^i)$, where $x^l$ is the center of compartment $l$, allows us to take the continuum limit $h\to 0$ of Equation (29) which gives the (S)PDE in Equation (4). The mean value of $N(I,t)$ can be written as $\langle N(I,t)\rangle = \sum_{i=m_1}^{m_2} hu(x^i,t)$, which is a Riemann sum. Taking the limit $h\to 0$ for constant $I$ gives

$$\langle N(I,t)\rangle \to \int_I dx u(x,t). \tag{22}$$

According to the *Countable additivity theorem*, the sum of an infinite number of Poisson distributed independent random variables converges with probability 1 if the sum of the mean values converges, and the sum has is Poisson distributed with corresponding mean value. We assume that the mean particle density is bound everywhere, which means that the values $u^i/h = u(x^i)$ are bound for all $i$ and all $h$. Let $B$ be such an upper bound. Since

$$\left|\sum_{i=m_1}^{m_2} hu(x^i)\right| \leq h \sum_{i=m_1}^{m_2} \left|u(x^i)\right| \leq h \sum_{i=m_1}^{m_2} B = h(m_2 - m_1)B, \tag{23}$$

the sum converges in the limit $h \to 0$ for constant $I = [(m_1 - \frac{1}{2})h, (m_2 + \frac{1}{2})h]$. We thus find that $N(I,t)$ is Poisson distributed in the continuum limit and we can write

$$P(N(I,t) = n) \xrightarrow{n\to\infty} \mathcal{P}(n; \int_I dxu(x,t)). \tag{24}$$

The same can be shown similarly for a countable union of subintervals of $[0,1]$, and $N(U_1,t)$ and $N(U_2,t)$ are obviously independent for disjunct $U_1$ and $U_2$. The probability distribution for any fixed $t$ is thus *exactly the same as the one of a spatial Poisson process* with intensity $u(x,t)$.

Suppose now the system also includes reactions of Type II. In this case the PR becomes stochastic, i.e., Equation (29) and its continuum version (4) contain non-vanishing noise terms. The field $u(x,t)$ is thus a random process. Given a realisation of $u(x,t)$, the same considerations as for the deterministic case apply and the single-time probability distribution behaves like a spatio-temporal Poisson process. Since $u(x,t)$ is now a random process, the single-time probability distribution of the system *corresponds exactly* to the one of a *spatial Cox process* with intensity $u(x,t)$. The same considerations hold in an approximate sense for Type III and IV reactions. These findings can easily be generalised to multiple-species systems and general spatial dimensions. This concludes the proof of Remark 1 and Result 1 in the main text.

# 2 Inference for Poisson and Cox processes

## 2.1 Numerical solution of (S)PDEs via basis projection

### General formulation

To apply the derived Cox process representation we need to solve (S)PDEs. We do this here approximately by means of a basis function projection leading to a finite set of coupled (stochastic) ordinary differential equations (SDEs/ODEs). For illustration we confine ourselves here to a one-dimension and one-species system, but the equations can be easily extended to multi-dimensional and multi-species systems. Consider an SPDE of the form

$$du(x,t) = A(x,t) + \sqrt{C(x,t)}dW(x,t), \tag{25}$$

where $A(x,t)$ and $C(x,t)$ are polynomials in $u(x,t)$ with potentially space-dependent coefficients. We approximate $u(x,t)$ by a linear-combination of a finite set of spatial basis functions $\{\phi_i(x)\}_{i=1}^n$,

$$u(x,t) = \sum_{i=1}^{n} c_i(t)\phi_i(x), \tag{26}$$

where we have introduced the time-dependent coefficients $c_i(t)$. Inserting this ansatz into (25), multiplying from the left with $\phi_j$ and integrating over $x$, it can be shown that the parameter vector $\mathbf{c} = (c_1, \ldots c_n)$ fulfils [1]

$$d\mathbf{c}(t) = \Phi^{-1}\langle\phi|A\rangle dt + \sqrt{\Phi^{-1}\langle\phi|C|\phi\rangle\Phi^{-1}}d\mathbf{W}, \tag{27}$$

where $d\mathbf{W}$ is a $n$-dimensional Wiener process and we have defined

$$\langle \phi_i | f \rangle = \int dx \phi_i(x) f(x,t), \tag{28}$$

$$\langle \phi_i | f | \phi_j \rangle = \int dx \phi_i(x) f(x,t) \phi_j(x), \tag{29}$$

$$\langle \phi | f \rangle_i = \langle \phi_i | f \rangle, \tag{30}$$

$$\langle \phi | f | \phi \rangle_{ij} = \langle \phi_i | f | \phi_j \rangle, \tag{31}$$

$$\Phi_{ij} = \langle \phi_i | \phi_j \rangle, \tag{32}$$

for a general function $f(x,t)$.

### For the real-valued Poisson representation

Due to the approximations of certain reaction types introduced in Section 1.2 the drift and diffusion terms in the SDE in (27) are always linear in the coefficient vector $\mathbf{c}$, with coefficients of the drift potentially depending on $\langle \mathbf{c} \rangle$, i.e., the drift may contain terms of the form $c_i \langle c_j \rangle$. Using this it is straightforward to show that the moment equations of $\mathbf{c}$ of different orders are not coupled to each other, i.e., the first-order moment equations depend only on first-order moments, etc. This in turn allows to directly numerically integrate the moment equations. Depending on the reactions involved, the diffusion term may be independent of $\mathbf{c}$ in which case the SDE in (27) has a multivariate Gaussian solution. The latter can be obtained by integrating the moment equations of up to order two. If the solution of the SDE is not Gaussian, we simply approximate it by a multivariate Gaussian with mean and variance obtained in the same way. Therefore, with the approximations introduced in Section 1.2, the SPDEs of all possible reaction systems can be solved by numerical solution of ODEs without the need for any additional approximations.

### Locally constant non-overlapping basis functions

We use locally constant, non-overlapping step functions throughout this work. For a one-dimensional system in the interval $[0, 1]$, for example, we define $n$ basis functions as

$$\psi(x) = \begin{cases} 1 & 0 \leq x \leq \frac{1}{n}, \\ 0 & \text{otherwise}, \end{cases} \tag{33}$$

$$\phi_i(x) = \psi(x - (i-1)/n) \quad \text{for} \quad i = 1, \ldots n. \tag{34}$$

The corresponding overlap and diffusion operator matrices read

$$\Phi = \langle \phi | \phi \rangle = \frac{1}{n} \mathbb{1}^{n \times n}, \tag{35}$$

$$\langle \phi | \Delta | \phi \rangle = n \begin{pmatrix} -1 & 1 & & & & & \\ 1 & -2 & 1 & & & & \\ & 1 & -2 & \ddots & & & \\ & & & \ddots & & & \\ & & & \ddots & -2 & 1 & \\ & & & & 1 & -2 & 1 \\ & & & & & 1 & -1 \end{pmatrix}, \tag{36}$$

where $\mathbb{1}^{n \times n}$ is the $n$-dimensional unity matrix and $\Delta$ is the Laplace operator.

## 2.2 Filtering

Here we describe the filtering procedure used to approximate likelihoods. Consider a Poisson process with intensity $u(x,t)$ given as the solution of a PDE as in (25) (with vanishing noise term), and spatial measurements $\mathbf{y} = (\mathbf{y}_{t_0}, \ldots, \mathbf{y}_{t_n})$ at discrete times $t_0, \ldots, t_n$. Suppose the intensity is approximated by a linear combination of basis function as in (26). Solving the PDE for $u(x,t)$ thus amounts to solving the system of ODEs in (27) (with vanishing noise terms) for the coefficient vector $\mathbf{c}$.

Since the intensity of a Poisson process is deterministic, the likelihood $p(\mathbf{y}|\Theta)$ of the data given the model $\Theta$ is simply computed by solving the ODE in $\mathbf{c}$ forward over the whole time interval and subsequently plugging in the measurements:

$$p(\mathbf{y}|\Theta) = \prod_{i=0}^{n} p(\mathbf{y}_{t_i}|\mathbf{c}(t_i)), \tag{37}$$

$$p(x_i|\mathbf{c}(t_i)) = \prod_{s \in x_i} u(s,t_i) e^{-\int dx u(x,t_i)}, \tag{38}$$

where $u(x,t_i)$ is given in terms of $\mathbf{c}(t_i)$ in (26).

In the case of a Cox process, the intensity $u(x,t)$ fulfils an SPDE and thus is a random process. After basis projection as in (27) the dynamics can be formulated in terms of the coefficients $c_i(t)$, which fulfil the system of SDEs in (27). As explained in Section 2.1, the latter is either solved by a Gaussian distribution or we approxiamte it by a Gaussian distribution. The likelihood has to be computed iteratively by solving the SDEs forward between measurement points and performing measurement updates. Suppose we have the Gaussian posterior $p(\mathbf{c}(t_{i-1})|\mathbf{y}_{t_{i-1}}, \ldots, \mathbf{y}_{t_0})$ at time $t_{i-1}$. Solving the SDE for $\mathbf{c}$ forward in time we obtain the predictive distribution $p(\mathbf{c}(t_1)|x_{i-1}, \ldots, x_0)$ which is again Gaussian. The posterior at time $t_i$ is then obtained by the Bayesian update as

$$p(\mathbf{c}(t_i)|\mathbf{y}_{t_i}, \ldots, \mathbf{y}_{t_0}) = \frac{p(\mathbf{y}_{t_i}|\mathbf{c}(t_i)) p(\mathbf{c}(t_i)|\mathbf{y}_{t_{i-1}}, \ldots, \mathbf{y}_{t_0})}{p(\mathbf{y}_{t_i}|\mathbf{y}_{t_{i-1}}, \ldots, \mathbf{y}_{t_0})}, \tag{39}$$

with likelihood contribution

$$p(\mathbf{y}_{t_i}|\mathbf{y}_{t_{i-1}}, \ldots, \mathbf{y}_{t_0}) = \int d\mathbf{c}(t_i) p(\mathbf{y}_{t_i}|\mathbf{c}(t_i)) p(\mathbf{c}(t_i)|\mathbf{y}_{t_{i-1}}, \ldots, \mathbf{y}_{t_0}), \tag{40}$$

where $p(\mathbf{y}_{t_i}|\mathbf{c}(t_i))$ is given in (38). The full likelihood is hence given by

$$p(\mathbf{y}|\Theta) = p(\mathbf{y}_{t_0}) \prod_{i=1}^{n} p(\mathbf{y}_{t_i}|\mathbf{y}_{t_{i-1}}, \ldots, \mathbf{y}_{t_0}). \tag{41}$$

The posterior in (39) is generally not Gaussian and intractable. We hence approximate it by a Gaussian using the *Laplace approximation* [1], which approximates the posterior by a Gaussian centred at the posterior's mode and with covariance being the negative Hessian of the posterior in the mode.

# 3 Details for studied systems

## 3.1 Gene expression

### Equations

Consider the gene expression system in Fig. 2 in the main text. For simplicitly, we consider a one-dimensional version here with the nucleus on one side of the cell. We do not model the

gene explicitly, but rather assume a homogeneous production of mRNA in the nucleus. The corresponding reactions are

$$\text{nucleus}: \quad \varnothing \xrightarrow{m_1} M, \tag{42}$$

$$\text{whole cell}: \quad M \xrightarrow{m_2} \varnothing, \tag{43}$$

$$\text{cytosol}: \quad M \xrightarrow{p_1} M + P, \tag{44}$$

$$\text{whole cell}: \quad P \xrightarrow{p_2} \varnothing, \tag{45}$$

and both the mRNA $M$ and protein $P$ diffuse across the whole cell with diffusion constants $d_M$ and $d_P$, respectively. After approximating the reaction in (44) as explained in Section 1.2 the PR for this system is real and deterministic, and we obtain using the SPDE in Equation (4)

$$du_M(x,t) = [d_M \Delta u_M(x,t) + m_1 h_n(x) - m_2 u_M(x,t)]dt, \tag{46}$$

$$du_P(x,t) = [d_P \Delta u_P(x,t) + p_1 h_c(x) u_M(x,t) - p_2 u_P(x,t)]dt, \tag{47}$$

$$h_n(x) = \frac{1}{r}\Theta(r-x), \tag{48}$$

$$h_c(x) = \frac{1}{1-r}\Theta(x-r), \tag{49}$$

where $r$ is the size of the nucleus and $\Theta$ the Heaviside step function. The functions $h_n(x)$ and $h_c(x)$ arise because $M$ and $P$ only become created in the nucleus and cytosol, respectively. If we additionally include the autocatalytic reaction

$$P \xrightarrow{p_3} P + P, \tag{50}$$

the equation for $u_P(x,t)$ becomes an SPDE and reads

$$du_P(x,t) = [d_P \Delta u_P(x,t) + p_1 h_c(x) u_M(x,t) + p_3 u_P(x,t) - p_2 u_P(x,t)]dt \tag{51}$$

$$+ \sqrt{2 p_3 u_P(x,t)}\, dW(x,t). \tag{52}$$

**Inference**

Consider first the system without the reaction in (50). In this case the system corresponds to a Poisson process. After basis function projection of the PDEs in (46) and (47) as explained in Section 2.1, we are left with solving a coupled system of ODEs and can compute data likelihoods as in (37). We fix the parameters to

$$r = 0.3, \quad d_M = 0.1, \quad m_1 = 20, \quad m_2 = 0.5, \quad d_P = 0.1, \quad p_1 = 20, \quad p_2 = 0.2. \tag{53}$$

We assume that initially there are zero mRNA molecules and zero protein molecules in the cell. We further assume that the mRNA is unobserved and consider measurements of the protein at thirty equally separated time points separated by $\Delta t = 0.5$. We project the PDEs in (46) and (47) onto twenty basis functions as explained in Section 2.1. We then optimise the likelihood of the data with respect to the parameters to obtain parameter estimates. We vary the initial values for the parameters in the likelihood optimiser randomly between 0.5 times and 2 times the exact value. The inference results are shown in Table 1 in the main text.

Next, we consider the same system but with the additional reaction in (50), for which the PDE in (47) gets replaced by the SPDE in (51). Now the system corresponds to a Cox process and we are left with solving a coupled system of SDEs after basis function projection. We approximate the solution of the SDEs by a multivariate Gaussian as described in Section 2.1. The corresponding likelihoods can then be computed as in (41). We again consider measurements of the protein at equally separated time points separated by $\Delta t = 0.5$ and optimise the corresponding likelihood. The results are shown in Table 2 in the main text.

## 3.2 SIRS

### Equations

The reactions of the SIRS system are

$$S + I \xrightarrow{k,w} 2I, \quad I \xrightarrow{r} R, \quad R \xrightarrow{s} S. \tag{54}$$

We consider a system in the two-dimensional square $[0,1] \times [0,1]$. After approximating the reaction in (44) as explained in Section 1.2 the PR for this system is real, and we obtain using Equation (4) for the intensity fields of $S$, $I$ and $R$,

$$du_S(x,t) = d\Delta u(x,t) - k^{\mathrm{PR}} u_S(x,t)u_I(x,t) + su_R(x,t), \tag{55}$$

$$du_I(x,t) = d\Delta u_I(x,t) + k^{\mathrm{PR}} u_S(x,t)u_I(x,t) - ru_I(x,t), \tag{56}$$

$$du_R(x,t) = d\Delta u_R(x,t) + ru_I(x,t) - su_R(x,t), \tag{57}$$

where we omitted noise terms in the equation for $u_I(x,t)$ for simplicitly and hence treat the system deterministically. We introduced the reaction rate $k^{\mathrm{PR}}$ in the term corresponding to the bimolecular infection reaction. If we include the additional spontaneous infection reaction

$$S \xrightarrow{v} I, \tag{58}$$

the equations for $u_S(x,t)$ and $u_I(x,t)$ obtain an additional term and read

$$du_S(x,t) = d\Delta u(x,t) - k^{\mathrm{PR}} u_S(x,t)u_I(x,t) + su_R(x,t) - vu_S(x,t), \tag{59}$$

$$du_I(x,t) = d\Delta u_I(x,t) + k^{\mathrm{PR}} u_S(x,t)u_I(x,t) - ru_I(x,t) + vu_S(x,t). \tag{60}$$

### Inference

As an initial condition we distribute $S_{ini}$ particles of species $S$ randomly across the whole area, one $I$ particle at $[0.05, 0.05]$ and assume zero $R$ particles. We simulate data for forty time points equally spaced by $\Delta t = 1$. As a basis we take 100 basis functions equally distributed in both dimensions. The inference results are shown in Table 3 in the main text.

## 3.3 Drosophila embryo

### Data and equations

The data of the Bicoid protein in Drosophila embryos used here consists of two-dimensional fluorescence data as depicted in Fig. 4a in the main text. Since the relation of measured fluorescence intensity to actual protein numbers is unknown we simply translate them one to one here. The Bicoid is typically modelled by a simple birth-death process with the reactions

$$\varnothing \xrightarrow{k_1} P, \quad P \xrightarrow{k_2} \varnothing. \tag{61}$$

For simplicity, since diffusion is radially symmetric, we only consider the data within a certain distance from the major axis of the embryos, thus effectively obtaining one-dimensional data. We assume further that the protein is produced within a certain range around the left tip of the embryos. Mathematically the system is thus equivalent to the mRNA system in Section 3.1. The intensity of the protein hence fulfils the PDE

$$du(x,t) = (d\Delta u(x,t) + k_1 f(x) - k_2 u(x,t))dt, \tag{62}$$

where $x$ is the distance from the left end of the embryo, $d$ is the diffusion constant, $k_1$ the production rate, $f(x) = 1, x \in [0,r], f(x) = 0, x \notin [0,1]$, $r$ is the production radius around the origin and $k_2$ is the decay rate.

### Inference

Since we have steady-state data, not all parameters are identifiable. One can easily see that multiplication of $k_1$, and $k_2$ with the same factor leads to the same steady state. We thus infer the creation range $r$, the diffusion rate $d$, and the ratio $c = k_2/k_1$. For the inference we project the PDE in (62) on twenty basis functions and solve the resulting ODEs for large times to ensure the solution to be in steady state. We optimise the likelihood for each of the embryos independently to obtain the inferred parameter values. The results are visualised in Fig. 4 in the main text.

# Supplementary References


[1] Zammit-Mangion, A., Dewar, M., Kadirkamanathan, V. & Sanguinetti, G. Point process modelling of the Afghan War Diary. *Proc. Natl. Acad. Sci. USA* **109,** 12414-12419 (2012).
